# Hog1 bypasses stress-mediated down-regulation of transcription by RNA polymerase II redistribution and chromatin remodeling

**DOI:** 10.1186/gb-2012-13-11-r106

**Published:** 2012-11-18

**Authors:** Mariona Nadal-Ribelles, Núria Conde, Oscar Flores, Juan González-Vallinas, Eduardo Eyras, Modesto Orozco, Eulàlia de Nadal, Francesc Posas

**Affiliations:** 1 Cell Signaling Unit, Departament de Ciències Experimentals i de la Salut, Universitat Pompeu Fabra (UPF), E-08003 Barcelona, Spain; 2 Joint IRB-BSC Program on Computational Biology, Institute for Research in Biomedicine, Baldiri i Reixac 10, 08028 Barcelona, Spain; 3 Computational Genomics, Universitat Pompeu Fabra, Dr Aiguader 88, E08003 Barcelona, Spain; 4 Catalan Institution for Research and Advanced Studies (ICREA), Passeig Lluís Companys 23, E08010 Barcelona, Spain

## Abstract

**Background:**

Cells are subjected to dramatic changes of gene expression upon environmental changes. Stress causes a general down-regulation of gene expression together with the induction of a set of stress-responsive genes. The p38-related stress-activated protein kinase Hog1 is an important regulator of transcription upon osmostress in yeast.

**Results:**

Genome-wide localization studies of RNA polymerase II (RNA Pol II) and Hog1 showed that stress induced major changes in RNA Pol II localization, with a shift toward stress-responsive genes relative to housekeeping genes. RNA Pol II relocalization required Hog1, which was also localized to stress-responsive loci. In addition to RNA Pol II-bound genes, Hog1 also localized to RNA polymerase III-bound genes, pointing to a wider role for Hog1 in transcriptional control than initially expected. Interestingly, an increasing association of Hog1 with stress-responsive genes was strongly correlated with chromatin remodeling and increased gene expression. Remarkably, MNase-Seq analysis showed that although chromatin structure was not significantly altered at a genome-wide level in response to stress, there was pronounced chromatin remodeling for those genes that displayed Hog1 association.

**Conclusion:**

Hog1 serves to bypass the general down-regulation of gene expression that occurs in response to osmostress, and does so both by targeting RNA Pol II machinery and by inducing chromatin remodeling at stress-responsive loci.

## Background

Yeast cells undergo major changes of gene expression in response to stress [1]. Global gene expression in response to osmostress in yeast has been studied in detail [2-9]. Major changes of gene expression occur in response to stress; many genes are down-regulated together with the up-regulation of a set of stress-responsive genes.

Activation of the high osmolarity glycerol (HOG) pathway upon stress regulates many aspects of cell physiology, including gene expression. The p38-related Hog1 stress-activated protein kinase (SAPK) is the master protein for reprogramming gene expression in response to osmostress through different specific transcription factors [5,7]. Hog1 is recruited to the osmoresponsive genes by these specific factors [10-17]. Once bound to chromatin, Hog1 serves as a platform to recruit RNA polymerase II (RNA Pol II) [13] and associated transcription factors [12,18-20]. Hog1 is present also at the coding regions of stress-responsive genes [15-17], where the kinase is essential for increased association of RNA Pol II and efficient mRNA production in response to osmostress [17]. Moreover, nucleosome positioning of specific stress-responsive loci is altered dramatically in a Hog1-dependent manner through the chromatin structure remodeling (RSC) complex upon osmostress [21].

Here, we assessed the genome-wide enrichment of RNA Pol II and Hog1 in response to stress by chromatin immunoprecipitation (ChIP) followed by sequencing (ChIP-Seq) as well as the re-organization of nucleosomes at stress-responsive loci by micrococcal nuclease followed by sequencing (MNase-Seq). We define a comprehensive picture of the genome-wide regulatory organization of the genome in response to stress and reveal Hog1 as the key protein needed to coordinate RNA Pol II relocalization, chromatin re-organization and osmospecific gene expression.

## Results and discussion

### Stress induces a rapid recruitment of RNA Pol II at stress-responsive loci

Analyses of gene expression have shown there is a rapid and strong induction of a set of stress-responsive genes in response to stress [2-9]. We quantified the increased fold induction of gene expression of 662 stress-responsive genes from microarray analysis (Materials and methods) and found an overall 6.4-fold increase of gene expression upon osmostress (0.4 M NaCl for 10 minutes). The induction pattern of these osmoresponsive genes in other stress conditions, such as heat shock (15 minutes at 37°C), oxidative stress (320 mM H_2_O_2_, 30 minutes), protein folding (250 mM dithiothreitol, 60 minutes) and amino acid starvation (30 minutes) [2] showed that osmoresponsive genes display a different expression pattern depending on each stress. In general, there is a poor overlap among the different stresses, with heat and osmostress overlapping the most (32%; Figure S1 in Additional file 1).

**Figure 1:**
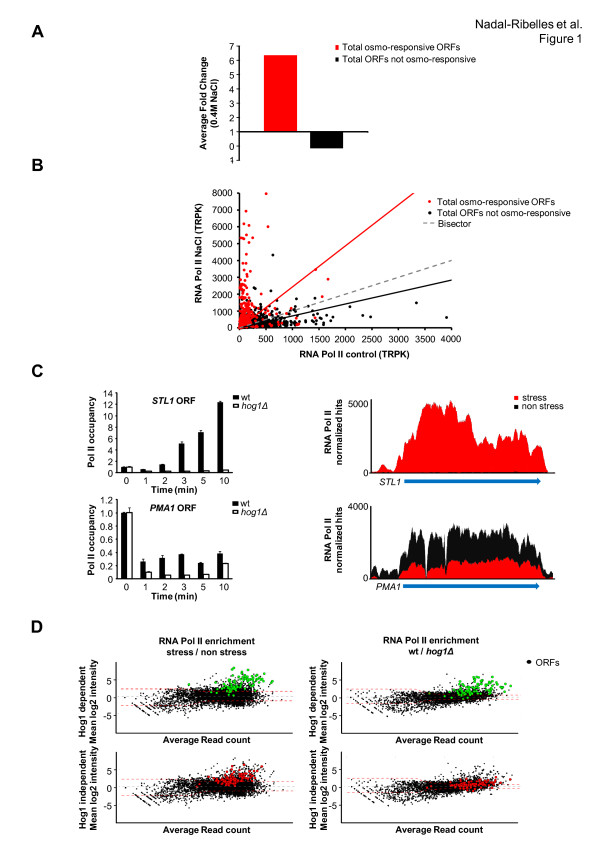
**Selective redistribution of RNA Pol II in response to osmostress**. **(a) **Comparison of gene expression changes from microarray data on wild-type (wt) cells subjected to osmostress (0.4 M NaCl, 15 minutes; see Materials and methods; Additional file 2). Bars represent the mean fold change for osmoresponsive ORFs with fold change > 1.75 upon osmostress (662 genes) and total ORFs present in the array except those considered osmoresponsive (5,655 genes; see Materials and methods). **(b) **A scatter plot showing RNA Pol II occupancy in basal conditions (YPD; x-axis) versus osmostress (0.4 M NaCl, 10 minutes; y-axis). Each dot represents normalized hits (TRPK; trimmed mean of *M *values normalized read/kilobase density). Black dots and the trend line represent TRPK distribution of total ORFs in the genome except osmoresponsive genes. Osmoresponsive genes are represented as red dots. **(c) **RNA Pol II binding kinetics to osmoresponsive and constitutively expressed genes. Left-hand panels: association of RNA Pol II upon osmostress (0.4 M NaCl, for the indicated times) was assessed by ChIP to *STL1 *osmoresponsive gene and *PMA1*. Real-time quantitative PCR (qPCR) results are shown as fold induction of treated versus non-treated (time zero). Right-hand panels: overlapping ChIP-Seq tracks representing RNA Pol II normalized hits at the *STL1 *and *PMA1 *loci in the presence (red histogram) or in the absence (black histogram) of osmostress. Red and black histograms have been overlaid. The blue arrow indicates annotated ORF. **(d) **Role of Hog1 in RNA Pol II recruitment. *MA *plots of RNA Pol II binding in wild-type upon osmostress (left-hand panel; see Materials and methods). *MA *plots of RNA Pol II binding wild-type versus *hog1 *mutant stressed as before. The dotted red line delimits the threshold for significance (*P *= 0.0001). Highlighted dots indicate a subset of 100 osmoresponsive genes that are differentially expressed based on the dependency of the SAPK (Hog1-dependent in green and Hog1-independent in red).

Whilst osmostress-induced genes showed a clear induction upon stress, the overall transcription of the whole genome, excluding the set of osmostress-induced genes, showed a 0.16-fold reduction in gene expression upon stress (Figure 1a). These data are consistent with previous reports [9] and indicate there must be a specialized mechanism that permits specific gene expression when global down-regulation of gene expression occurs.

To characterize how the changes of gene expression in response to osmostress are accomplished, we analyzed genome-wide binding of RNA Pol II in response to osmostress by ChIP-Seq in wild-type and *hog1 *cells. Association of RNA Pol II with ORFs is reduced when the overall genome is considered, whereas it clearly increases for stress-responsive genes (Figure 1b). Earlier studies showed that some housekeeping genes suffered a strong reduction of RNA Pol II occupancy at early time points in response to stress [22]. This is exemplified by the increase of RNA Pol II at the *STL1 *osmoresponsive gene in contrast to the reduction of overall RNA Pol II observed at the housekeeping gene *PMA1*, which encodes an essential H-ATPase (Figure 1c). Thus, *STL1 *and *PMA1 *genes are clear examples that represent the trend in osmoresponsive versus housekeeping genes. It also shows that while RNA Pol II is lost at the housekeeping genes in both wild-type and *hog1 *strains, the wild-type strain shows a faster recovery of RNA Pol II. Of note, the down-regulation of RNA Pol II in house-keeping genes precedes the recruitment of RNA Pol II at stress-responsive genes, indicating that the overall reduction of RNA Pol II occupancy cannot be due to a decrease in its availability. Taken together, genome-wide RNA Pol II localization suggests a strong bias for its localization towards stress-responsive genes.

Stress-responsive genes can be classified into two groups, Hog1-dependent and Hog1-independent, on the basis of gene expression data (Materials and methods; Additional file 2). When the 100 most responsive genes of each group were analyzed we found that indeed there was a clear difference in the degree of induction; the Hog1-dependent genes displayed a fold change that was almost four times higher compared to Hog1-independent genes (Figure S2 in Additional file 1). We then analyzed each group with regard to RNA Pol II association and found that RNA Pol II was recruited significantly to Hog1-dependent (green dots) and Hog1-independent (red dots) responsive genes in response to stress (Figure 1d, left-hand panels). By contrast, recruitment of RNA Pol II to Hog1-dependent genes was significantly different between wild-type and *hog1 *strains (green dots), while no differences were observed between a wild-type and a *hog1 *strain with regard to Hog1-independent genes (red dots) (Figure 1d, right-hand panels). The association of RNA Pol II with down-regulated genes or housekeeping genes in a wild-type strain was similar to that in a *hog1 *strain, indicating that Hog1 does not play a role in the initial changes observed in non-stress-dependent genes (Figure S3 in Additional file 1). Thus, Hog1 has a crucial role in the genome-wide redistribution of RNA Pol II to stress-responsive genes upon stress.

### Hog1 associates with the chromatin of RNA Pol II and Pol III genes

Genome-wide studies using ChIP and microarray analysis have been instrumental in uncovering the presence of Hog1 associated with a number of stress-responsive genes as well as its localization at both promoter and coding regions of stress-responsive genes. However, the number of genes uncovered by these approaches has been rather limited and never totaled more than 70 genes [15-17]. The relevance of Hog1 in gene expression and RNA Pol II recruitment suggested that the number of genes with Hog1 association could have been underestimated. We undertook ChIP-Seq analysis to improve the sensitivity of detection and found that Hog1 is present in at least 340 genome loci upon osmostress (0.4 M NaCl for 5 minutes; Figure S4a in Additional file 1). We analyzed binding at 5 minutes because this was the peak of Hog1 association with *STL1 *and *CTT1 *[19] (Figure S5 in Additional file 1). Albeit ChIP experiments generate data that are population averages, previous single cell analyses showed that Hog1 is activated in all cells similarly upon osmostress and that transcriptional induction correlates very well with the localization of Hog1 to stress-responsive genes [23]. Recruitment of Hog1 was not restricted to RNA Pol II transcribed genes but was present, albeit to a lesser extent, at RNA Pol III transcribed genes as well as long terminal repeat (LTR) DNA regions.

**Figure 2:**
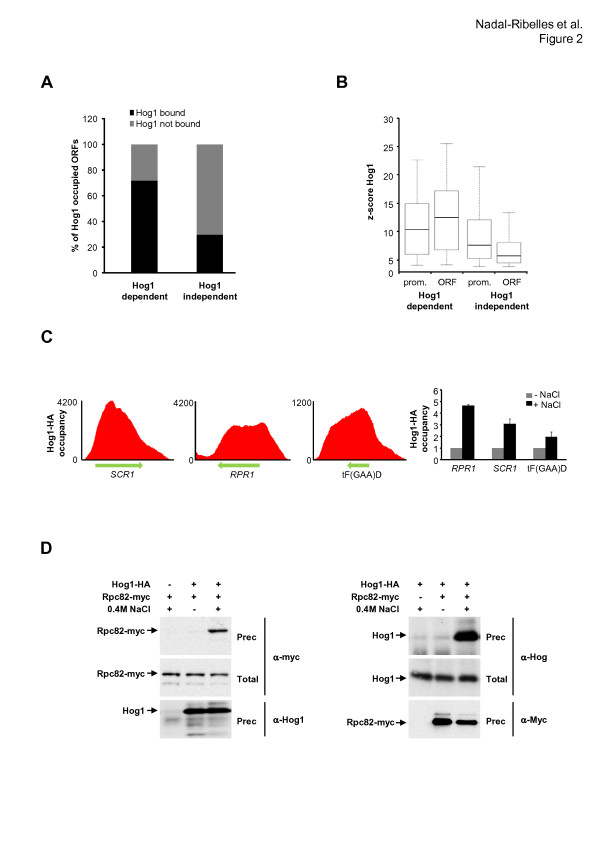
**Hog1 associates with RNA Pol II and RNA Pol III loci upon stress**. **(a) **Stacked bars representing the percentage of Hog1-occupied ORFs upon stress as determined by ChIP-Seq data for Hog1-dependent and -independent subsets of genes (*Z*-score 4; *P *= 0.0001), as in Figure 1d). **(b) **Boxplot of Hog1 association upon stress (*Z*-score) at promoters (from -500 to the transcription start site) and ORFs in genes whose expression is classified as Hog1-dependent or Hog1-independent. **(c) **Hog1 recruitment at RNA Pol III loci of *SCR1*, *RPR1 *and tF(GAA)D. Histograms represent Hog1 ChIP-Seq profiles from a non-stressed sample normalized to that obtained upon osmostress. Right-hand panel: Validation of Hog1 recruitment to novel Hog1 sites by ChIP. **(d) ***In vivo *binding of Hog1 and RNA Pol III. Hog1-HA and a specific subunit of RNA Pol III (Rpc82-Myc) were expressed from their endogenous locus. Samples were taken before (-) and after (+) stress (0.4 M NaCl, 10 minutes).

When the presence of Hog1 was analyzed on RNA Pol II transcribed genes, we found that Hog1 was associated with approximately 80% of genes, with expression described to be highly dependent on the SAPK, confirming that Hog1 is widely associated with Hog1-regulated genes. By contrast, only 30% of the genes induced upon osmostress are Hog1-independent and showed Hog1 associated with their loci (Figure 2a; Figure S4b in Additional file 1, green and red dots). Hog1 was not present at down-regulated or house-keeping genes (Figure S4b in Additional file 1, blue and yellow dots); therefore, Hog1 is associated with stress-responsive RNA Pol II genes.

Hog1 has been shown to associate with promoters and the ORFs of stress-responsive genes. We asked whether there was a biased interaction towards promoters or ORFs and found that Hog1 was associated with both promoters and ORFs in a significant number of genes (more than 41%). Also, for those genes where only one region was above the threshold, association of Hog1 with ORFs was more prominent than with promoters (Figure S6 in Additional file 1). When we analyzed association of Hog1 with Hog1-dependent or Hog1-independent genes, we found that Hog1 binding is biased slightly towards the ORFs in Hog1-dependent genes, whereas for genes for which Hog1 was less relevant, Hog1 localization was associated mainly with promoters (Figure 2b). Several scenarios could explain the presence of the SAPK at Hog1-independent loci, such as the use of a too stringent threshold for Hog1 dependency, their presence close to a Hog1-dependent gene, or the induction of the gene is mediated by redundant pathways, including Hog1. Thus, stress-induced RNA Pol II transcribed genes appear to have strong enrichment of Hog1 at their promoters and ORFs.

Remarkably, Hog1 was also associated with RNA Pol III transcribed genes, including at least 16 tRNA genes as well as the two reference genes *SCR1 *and *RPR1 *(Figure 2c). ChIP experiments showed similar kinetics of association of Hog1 with tF(GAA)D, *RPR1 *and *SCR1 *as with RNA Pol II transcribed genes. We then investigated the association of RNA Pol III (Rpc82 subunit) with two tRNA loci (tF(GAA)D and tP(UGG)O3) and found that, albeit RNA Pol III disassociated rapidly from chromatin in a stress-dependent manner, a rapid recovery of RNA Pol III levels occurred in wild-type that was not observed in *hog1 *cells (Figure S7 in Additional file 1).

It has been reported that Hog1 interacts with RNA Pol II (most likely through Rpb1), which facilitates gene expression in RNA Pol II transcribed genes [13,17]. Therefore, we used co-precipitation experiments in extracts from cells expressing endogenously tagged HA-Hog1 and Myc-Rpc82 (a subunit of the RNA Pol III complex not shared with RNA Pol II) to assess whether Hog1 is able to interact with RNA Pol III. We found Hog1 was able to interact with endogenous tagged-Rpc82 and vice versa (Figure 2d). It is worth noting that this interaction was observed only when cells were subjected to osmostress. Thus, Hog1 is targeted to RNA Pol III loci in response to stress and associates physically with RNA Pol III, as it does with RNA Pol II transcribed genes.

### Efficient recruitment of RNA Pol II and maximal gene expression requires Hog1

**Figure 3:**
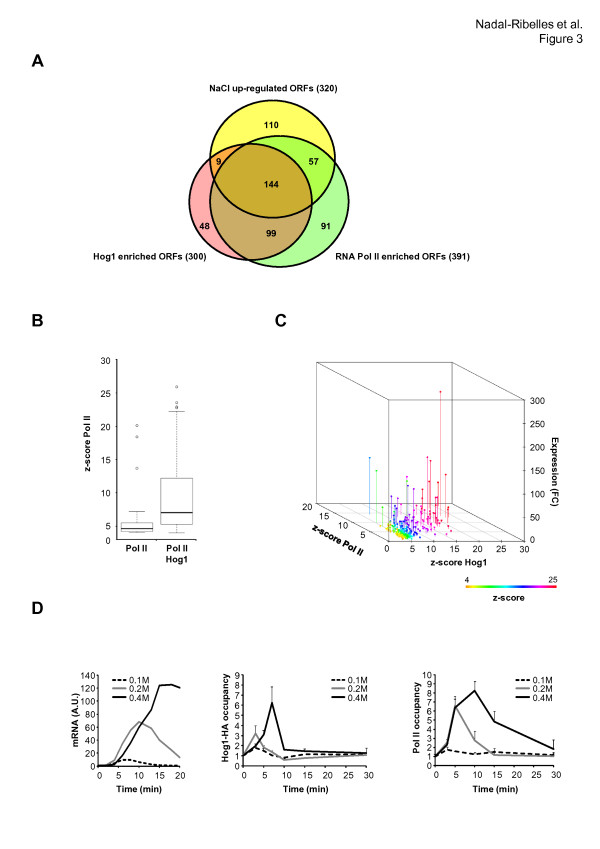
**Greater enrichment of Hog1 induces stronger recruitment of RNA Pol II and determines transcriptional output**. **(a) **A Venn diagram representing the overlap of Hog1-bound genes (*Z*-score > 4) and recruited RNA Pol II (*Z*-score > 6) with 320 representative genes that are up-regulated upon osmostress by more than three-fold. **(b) **Colocalization of Hog1 with RNA Pol II results in stronger binding of both to chromatin. The boxplot represents distribution of *Z*-score values of genes with Hog1 enrichment and RNA Pol II compared to those genes with RNA Pol II but without Hog1 association. **(c) **Positive correlation between Hog1, RNA Pol II binding and expression in response to stress. Each point in the three-dimensional graph represents the 144 genes (overlapping region from (a)); the x-axis represents binding of Hog1, the y-axis binding of Rbp1 (both as *Z*-score values) and the z-axis the expression as the fold change (*FC*) of a wild-type strain upon stress. **(d) **Gene expression is dependent on the duration and intensity of Hog1 binding to target genes. The dose-response expression of osmoresponsive genes was assayed by northern blot and probed for *STL1 *(left-hand panel). Association of tagged Hog1-HA (middle panel) and Rpb1 (right-hand panel) to the promoter region of *STL1 *was analyzed by ChIP. The results are shown as the fold induction of stressed against non-stressed (time zero) cultures.

To assess the relevance of the association of Hog1 with RNA Pol II, we compared the degree of gene expression of stress-responsive genes with the presence of RNA Pol II and Hog1 (Figure 3a). Several groups of osmoresponsive genes can be identified, depending on the presence of Hog1 and/or RNA Pol II upon stress. A group of genes showed no significant association of Hog1 with RNA Pol II but were induced upon osmostress. This group of genes correlated quite well with genes that showed stabilization of mRNAs upon stress (40 out of the 43 analyzed were found to be stabilized) [8,9]. We found a significant number of genes with increased RNA Pol II association that did not have Hog1 present on them (91 out of 391); these genes correspond to Hog1-independent genes. There was, however, a prominent overlapping group of genes that showed increased expression, increased recruitment of RNA Pol II and association with Hog1 (a total of 144 genes; Figure 3a). We then compared the degree of gene induction in those groups of genes and found that there was a strong correlation between the presence of both Hog1 and RNA Pol II with high expression rates when compared to genes that were enriched only with RNA Pol II (Figure 3b). If the presence of Hog1 improved the recruitment of RNA Pol II and transcription, it should be possible to establish a quantitative relationship between weak, or strong, Hog1 binding with RNA Pol II and transcription. Co-localization studies of Hog1 with RNA Pol II showed that RNA Pol II association with stress-responsive genes was more efficient for genes with higher Hog1 association (Figure 3c). Therefore, a high level of induction in stress-responsive genes is accomplished by strong association with Hog1 and increased RNA Pol II recruitment.

It has been reported that very low salt (0.1 M NaCl) stress results in maximal Hog1 activation. When cells are exposed to higher concentrations of NaCl, however, activation of Hog1 remains associated with stress-responsive loci for an extended period of time [19,23]. If the presence of Hog1 improves the recruitment of RNA Pol II and transcription efficiency, it would be expected that an increase of Hog1 at specific promoters will result in enhanced recruitment of RNA Pol II and transcription. We followed *STL1 *expression in response to 0.1, 0.2 and 0.4 M NaCl together with the association of Hog1 and RNA Pol II. Weak expression of *STL1 *was observed at 0.1 and 0.2 M NaCl, in clear contrast to the induction observed with 0.4 M NaCl (similar results were observed for *CTT1 *and *ALD3*). Remarkably, the initial recruitment of RNA Pol II at *STL1 *was similar at 0.2 and 0.4 M NaCl; however, the residence time of Hog1 at the loci was clearly shorter (Figure 3d; Figure S8 in Additional file 1). Thus, the association time of Hog1 with stress loci appears to be crucial for determining the degree of gene induction upon osmostress.

### Hog1 mediates chromatin changes at stress-responsive loci

**Figure 4:**
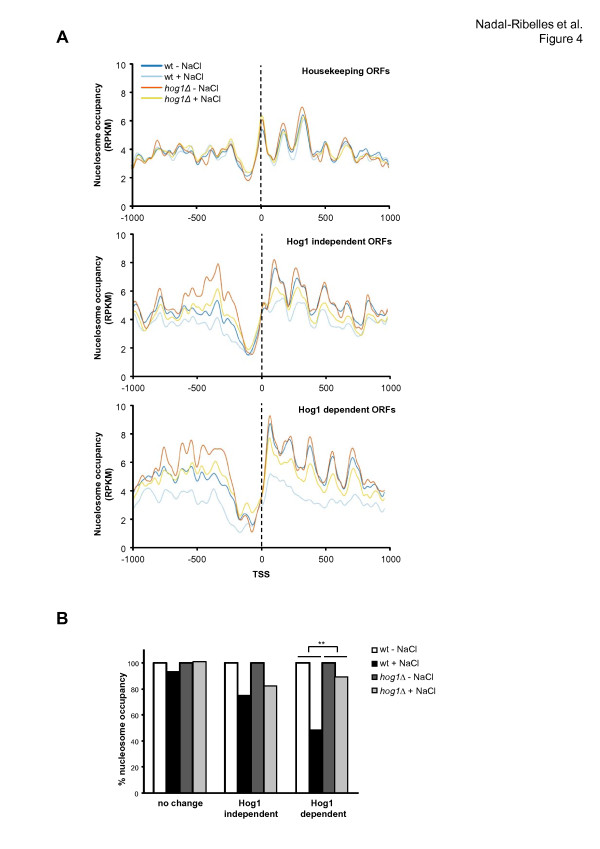
**Hog1 mediates major changes in chromatin structure to facilitate transcription of stress-responsive genes**. **(a) **The distribution of nucleosomes referenced as reads/kilobase per million mapped reads (RPKM; transcription start site (TSS) ± 1 kb) of wild-type (wt; blue) and *hog1 *mutant (red) strains subjected (or not) to osmostress as indicated in the key. The plot represents the mean of reads in a group of 100 genes without transcription changes upon stress (upper panel), 100 genes that are induced in a Hog1-independent manner (middle panel) and 100 genes whose expression is induced upon osmostress depending on the SAPK (lower panel) (as in Figure 1d). The dotted black line marks the TSS. **(b) **The percentage of nucleosome occupancy in subsets from (a). Nucleosome occupancy was determined by averaging the TRPKs (trimmed mean of *M *values normalized read/kilobase density) from the 200 bp immediately downstream of the TSS. Average reads of non-stressed samples was used as maximum occupancy and as a reference for treated samples. **The statistical significance of the difference was assessed by a paired Student *t*-test of acceptance of equality at (*P*-value < 0.01) comparing the eviction of wild-type versus *hog1 *upon stress.

Hog1 stimulates chromatin remodeling at specific stress-responsive loci by recruiting the RSC chromatin remodeler [21]. We investigated whether all stress-responsive genes were subjected to changes on chromatin organization and the relevance of the SAPK to those changes. We used genome-wide MNase digestion of chromatin and deep sequencing (MNase-Seq) before and after stress. Wild-type and *hog1 *strains were subjected (or not) to osmostress and cells were fixed before digestion of chromatin by MNase to prevent Hog1 activation during the preparation of spheroplasts (see Materials and methods). The nucleosomal profile around the transcription start site (TSS) in genes that were not regulated upon stress did not change when cells were subjected to osmostress (Figure 4a, upper panel). We then analyzed nucleosome positioning in stress-induced genes with expression that does not depend on Hog1. Upon osmostress there were slight changes of nucleosome occupancy, especially around the TSS and those changes were similar in *hog1*-deficient cells (Figure 4a, middle panel). In clear contrast, when Hog1-dependent genes were analyzed, a dramatic change of nucleosome occupancy occurred upon stress at both the promoter and ORF regions. These changes on nucleosomes were completely abolished in *hog1 *cells. It is noteworthy that the +1 nucleosome in stress-responsive genes appears to be shifted slightly when compared to localization of the genome-wide +1 nucleosome, suggesting a particular chromatin structure for stress-responsive genes. Color maps of -1,000 to +1,000 of each ORF aligned by TSS have also been included to illustrate nucleosome organization genome-wide and in stress-responsive genes (Figure S9 in Additional file 1). Taken together, efficient nucleosome re-organization at stress-responsive genes is completely dependent on the SAPK (Figure 4a, lower panel). When changes on the chromatin structure were quantified (percentage of nucleosome occupancy), we found that osmostress induces a 25% decrease in nucleosome occu-pancy in Hog1-independent genes (similar to that found in wild-type and *hog1 *cells), whereas it was decreased 51% in Hog1-dependent genes (Figure 4b). Thus, Hog1 is crucial to inducing major changes in chromatin structure.

In summary, genome-wide binding studies in combination with analysis of chromatin structure have shown that Hog1 serves to bypass the general down-regulation of gene expression that occurs in response to stress. Hog1 permits efficient targeting of the RNA Pol II machinery and, furthermore, induces major changes of chromatin structure at stress-responsive loci. The combination of targeted recruitment of RNA Pol II with chromatin remodeling is essential to maximize gene expression in response to external stimuli.

## Conclusions

In response to an environmental insult such as osmostress, there are major changes of RNA Pol II localization towards stress-responsive genes, in contrast to housekeeping genes, which correlates with down-regulation of general transcription. This indicates that while there is general down-regulation of expression and RNA Pol II association with housekeeping genes, RNA Pol II is recruited to stress-responsive genes. We showed that this RNA Pol II relocalization is a phenomenon that requires the Hog1 SAPK. We analyzed genome-wide localization of the SAPK and found its presence in Hog1-dependent genes more frequently than in any published study and, remarkably, we found Hog1 associated with RNA Pol III genes, showing for the first time the interaction of the SAPK with the RNA Pol III machinery. We undertook genome-wide analysis of the change of chromatin structure upon stress. We showed that there is strong remodeling of chromatin restricted to stress-responsive genes, which depends completely on the SAPK. Hog1 association and chromatin remodeling correlated very well with higher levels of transcription, which highlights the relevance of chromatin regulation and transcription.

Hog1 serves to bypass the general down-regulation of gene expression that occurs in response to osmostress by targeting RNA Pol II machinery and by inducing chromatin remodeling at stress-responsive loci.

## Materials and methods

### Yeast strains and plasmids

*Saccharomyces cerevisiae *strain BY4741 (MATa *his3*-Δ*1 leu2*-Δ*0 met15*-Δ*0 ura3*-Δ*0*) and its derivatives YGM61 (*HOG1*::*KanMX4*), the carboxy-terminal tagged strain YEN173 (Hog1-6HA::*HIS3*), YMN07 (Rpc82-18myc::*KanMX4*), YMN10 (Rpc82-18myc:: *KanMX4 HOG1*::*URA3*) and YMN14 (Rpc8218myc::*KanMX4 *Hog1-6HA::*HIS3*) were used. Epitope tagging or gene deletions were done with a PCR-based strategy.

### ChIP-Seq

Wild-type and *hog1 *mutant (YGM61) *S. cerevisiae *strains were grown to early log phase and exposed to osmostress (0.4 M NaCl) for 5 minutes for Hog1 immunoprecipitation (anti-HA 12CA5) and for 10 minutes for RNA Pol II immunoprecipitation (8WG16, Covance, Richmond, CA, USA). ChIP was done as described [10,24]; 10 ng of DNA from each ChIP and condition was used to create sequencing libraries and then subjected to 36-nucleotide single-read sequencing on a Solexa Genome Analyzer IIx instrument. Only sequencing reads that mapped to only one location were aligned to the *S. cerevisiae *genome (sacCer2) allowing up to three mismatches/reads. Chip reads were extended by 250 bp and normalized using Pyicos software [25]. Enrichment of Hog1 and RNA Pol II was done by running the Pyicos enrichment protocol [25] comparing untreated to treated samples. Hog1-dependent RNA Pol II recruitment was determined by comparing salt-treated samples with wild-type and *hog1 *strains. For all comparisons, enrichment was considered significant for a *Z*-score > 4 (*P *= 0.0001). *MA *plots were done using the Pyicos software, where *M *represents the log ratio of stressed versus non-stressed (y-axis) and *A *is the average of the log intensities (x-axis) of all the genes, for enrichment of Hog1 and RNA Pol II. *MA *plots for Hog1-dependent RNA Pol II enrichment (Figure 1d, right-hand panels) compare NaCl-treated samples in a wild-type versus *hog1 *strain. ChIP-Seq data have been deposited at the NCBI Gene Expression Omnibus (GEO) database with accession number GSE41494.

### MNase nucleosome mapping

Wild-type and *hog1 *mutant (YGM61) strains were grown to early log phase in YPD medium, then subjected (or not) to osmostress (0.4 M NaCl, 10 minutes). Spheroplasts and digestion with MNase were done essentially as described but with some modifications [21]. Spheroplasts were prepared from mid-log phase cultures grown in YPD medium, following crosslinking with 1% (v/v) formaldehyde for 20 minutes, treated with 125 mM glycine for 15 minutes and washed four times with Tris-buffered saline (20 mM Tris-HCl pH, NaCl 150 mM). Cells were then lysed and immediately digested with 7.5 to 125 mU of MNase (Worthington Biochemical Corporation, Lakewood; NJ., USA). DNA was subjected to electrophoresis in a 1.5% (w/v) agarose gel and then stained with ethidium bromide. The band corresponding to the mononucleosome was cut from the gel and purified using a QIAquick gel extraction kit (Qiagen, Chatsworth, CA, USA). Mononucleosomal DNA from each sample used to create sequencing libraries was subjected to 36-nucleotide single-read sequencing on a Solexa Genome Analyzer IIx. Sequencing reads were aligned to the *S. cerevisiae *reference genome using Bowtie software [26] allowing up to three mismatches/reads. Over-represented reads were eliminated to reduce PCR amplification artifacts. Coverage values were calculated for each position on the genome, normalized and converted to reads per million. The mean reads per million was obtained by aligning genes to the TSS. The TSS used was obtained from the 'saccer2' database available from the Saccharomyces Genome Database (2008). All the annotated features, including the TSS, come from the Saccharomyces Genome Database annotation. Peak detection was done with the *nucleR *software package [27]. The percentage of nucleosome occupancy was determined by running an extension and normalization protocol using the first 200 bp downstream of the TSS, which encompasses the +1 nucleosome for all the genes clusters, using Pyicos software [25]. Eviction was determined by setting the non-treated nucleosome occupancy in TRPK (trimmed mean of *M *values normalized read/kilobase density) at time zero of each strain as the maximum occupancy (100%) and used as the reference for treated samples. MNase-Seq data have been deposited at the NCBI GEO site with accession number GSE41494.

### Chromatin immunoprecipitation

Yeast cultures were grown to early log phase, then subjected to osmostress at different salt concentrations (0.1 M, 0.2 M or 0.4 M NaCl); ChIP was done as described [13]. Antibodies used for immunoprecipitation were anti-HA 12CA5 for Hog1-HA, 8WG16 (Covance) for Rpb1 or anti-myc, 9E10 for Rpc82-Myc. For crosslinking, yeast cells were treated with 1% formaldehyde for 20 minutes at 25°C. Conventional and real-time PCR analysis of stressed and constitutively expressed genes used the following primers with locations indicated by the distance from the respective ATG initiation codon: *STL1 *(promoter, -372/-112; ORF, 1,475/1,575), *CTT1 *(promoter, -432/-302; ORF, 736/836), *PMA1 *(+1,010/+1,250), *SCR1 *(+19/+369), *RPR1 *(+100/+325), tP(UGG)O3 (-61/+185) and tF(GAA)D (-74/+226) and *TEL *(telomeric region on the right arm of chromosome VI). Experiments were done with three independent chromatin preparations and quantitative PCR analysis was done in real time with a sequence detector (ABI 7700, Applied Biosystems, Foster City, CA., USA). Immunoprecipitation efficiency was calculated in triplicate by dividing the amount of PCR product in the immunoprecipitated sample by that in the *TEL *sequence control. The binding data are presented as fold induction with respect to the non-treated condition.

### Northern blot analysis

Yeast cultures were grown to early log phase (absorbance at 660 nm 0.6 to 0.8). Cells were subjected (or not) to 0.1 M, 0.2 M or 0.4 M NaCl. Total RNA was probed by using radiolabeled fragments *ALD3 *(1.5 kb), *STL1 *(1.7 kb), *CTT1 *(1.7 kb) and *ENO1 *(1.3 kb). Signals were quantified with a phosphorimager (Typhoon 8600, Molecular Dynamics, Sunnyvale, CA., USA) using ImageQuant software (Molecular Dynamics, Sunnyvale, CA., USA).

### *In vivo *co-precipitation assay

Rpc82 and/or Hog1-tagged cells in mid-log phase were treated (or not) with 0.4 M NaCl for 15 minutes and then collected extracts were subjected to immunoprecipitation as described [20] with anti-HA 12CA5 or anti-Myc 9E10. Proteins were detected by western blotting against Hog1 (anti-Hog1, Santa Cruz Biotechnology, Santa Cruz, CA,, USA) and anti-Myc 9E10.

### Gene expression studies

Microarray experiments and data analysis were done as described [28]. Briefly, three independent cultures of wild-type strains were grown to exponential phase in YPD medium and stressed (or not) with 0.4 M NaCl for 15 minutes. Gene expression was determined by comparing the expression of the non-stressed versus stressed conditions. RNA microarray data have been deposited at the NCBI GEO with accession number GSE41451. On the bases of our microarray data together with the results of earlier studies [3], we have created different gene clusters depending on their expression under osmostress and their dependence on the presence of Hog1. The 'osmoresponsive genes' are a group of 662 ORFs whose fold change (FC) upon stress is > 1.75 in a wild-type stain (microarray data from this study), and the 'NaCl group' contains 320 genes with FC > 3 (Figure 1a and 2a, respectively). From these mentioned groups, we chose a subset of 100 genes whose expression is considered Hog1-dependent or Hog1-independent with the following criteria: 'Hog1-dependent genes' are those genes whose expression depends at least 25% on the presence of the mitogen-activated protein kinase (MAPK) described in [3]. 'Hog1-independent genes' are those genes whose expression in a *hog1 *strain is at least 90% similar to the wild-type strain described in [3]; moreover, genes that showed enrichment for Hog1 at their coding regions but their expression was not considered Hog1-dependent have been considered independent. The 'housekeeping genes' are those genes whose expression upon osmostress remained unchanged (FC 1 to 1.1). The 'down-regulated genes' are those genes whose expression was at least two-fold lower upon osmostress. See Additional file S2 for the complete list of genes included in each category.

## Abbreviations

ChIP: chromatin immunoprecipitation; FC: fold change; GEO: Gene Expression Omnibus; HOG: high osmolarity glycerol; MNase: micrococcal nuclease; ORF: open reading frame; Pol: polymerase; SAPK: stress-activated protein kinase; TSS: transcription start site.

## Competing interests

The authors declare that they have no competing interests.

## Authors' contributions

MN and NC did most of the experiments and analysis. OF, MO, JG and EE did the bioinformatics analysis. MN, NC, EN and FP did the experimental designs and wrote the paper. All authors have read and approved the manuscript for publication.

## Supplementary Material

Additional file 1 **Supplementary Figure S1 to S9**. Click here for file

Additional file 2 **Supplemetary Table 1**. List of all the genes considered in the manuscript as Hog1-dependent (top 100 Hog1-depedent osmoresponsive genes), Hog1-independent (top 100 Hog1-independent osmoresponsive genes) and a list of all osmoresponsive genes from the microarray analysis (see the 'Gene expression studies' section in Materials and methods for the criteria used to define the genes present in the list). Click here for file
