# Supplementary material for: Hog1 bypasses stress-mediated down-regulation of transcription by RNA polymerase II redistribution and chromatin remodeling
Source: Genome Biol. 2012 Nov 18;13(11):R106. doi: 10.1186/gb-2012-13-11-r106 (PMC3580498; doi:10.1186/gb-2012-13-11-r106)
Supplement: Additional file 1 — Supplementary Figure S1 to S9. [file gb-2012-13-11-r106-S1.PDF]

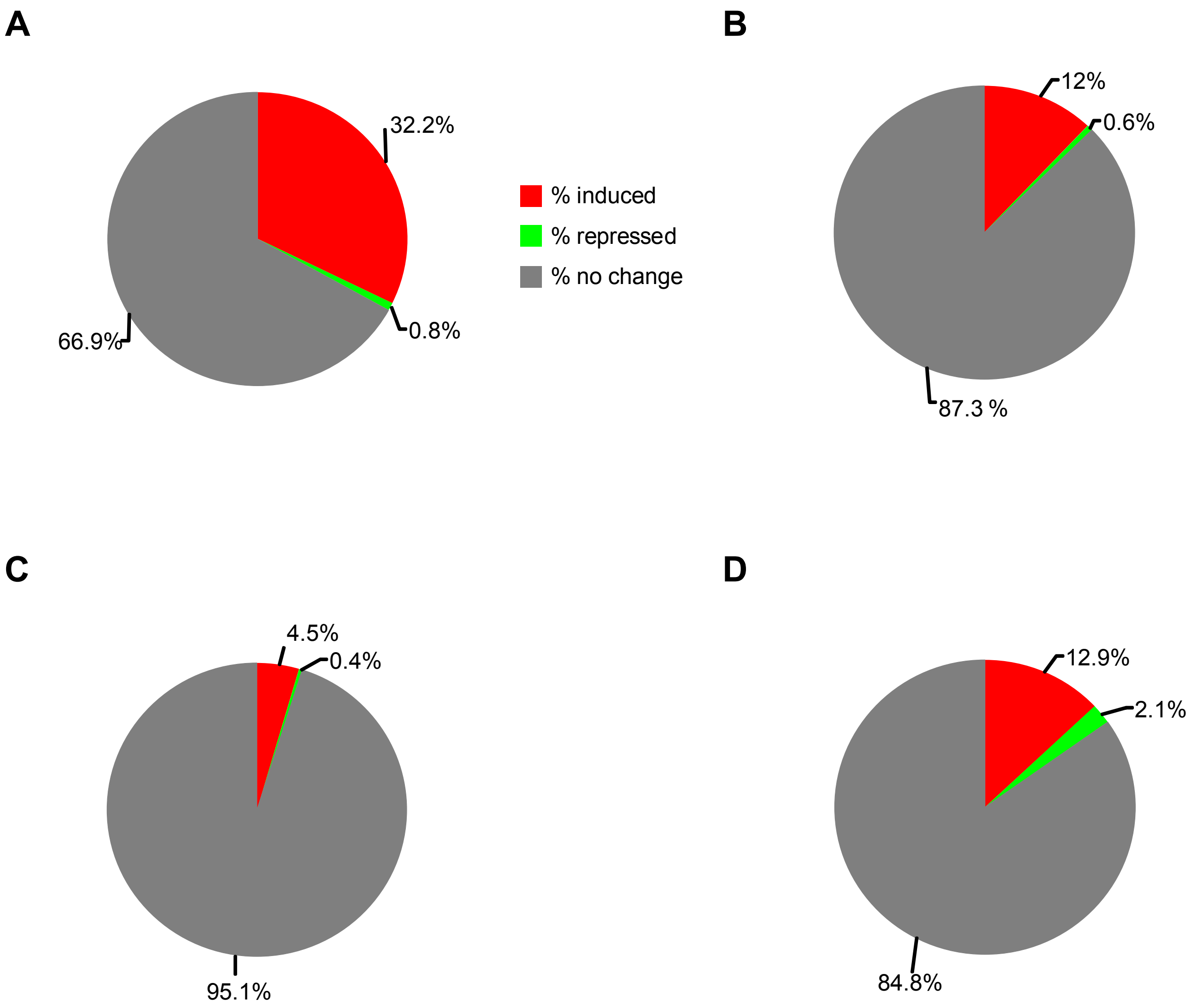

Fig S1 | Expression of osmo-responsive genes remains mostly unchanged upon other environmental stresses. Total osmo-responsive genes (662) were compared to available expression arrays with different environmental stresses (Gasch *et al*., 2000) such as **(A)** heat stress (15 minutes at 37ºC), **(B)** oxidative stress (320 mM H_2O_2 for 30 minutes), **(C)** DTT (250 mM for 60 minutes) and **(D)** aminoacid starvation (30 minutes). Genes were determined to be induced (FC>2), repressed (FC<-2) or unchanged if fold change remained within the induced/repressed threshold upon stress. These genes are shown in red, green and grey respectively together with the percentage from the total genes considered.

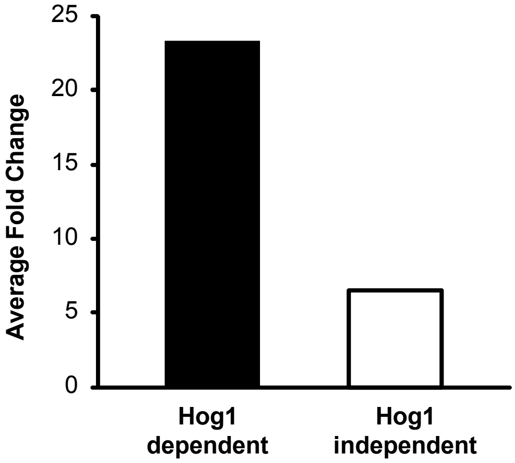

Fig S2 | Hog1-dependent genes have higher expression than Hog1-independent genes upon osmostress. Comparison of average fold change upon stress of Hog1-dependent (black) and independent (white).

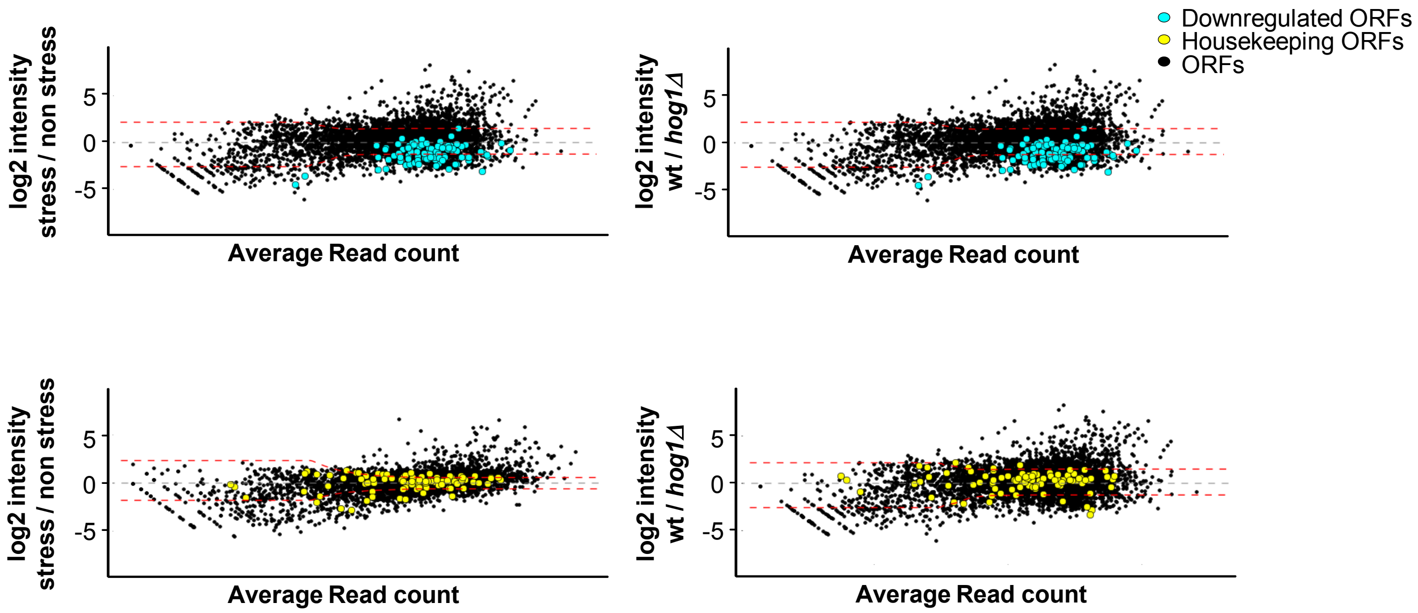

Fig S3 | MA Plots representing RNA Pol II in a subset of 100 genes (blue) whose expression is down-regulated upon more than two fold upon osmostress and a group of 100 genes (yellow) whose fold change upon stress remained unchanged (FC 1 to 1.1) Left panels show wild type RNA Pol II recruitment while right panels represent Hog1-dependent recruitment of RNA Pol II.

A

	Hog1 enrichment	region detail
**RNA Pol II**	300	
**RNA Pol III**	18	16 tRNAs<br>1 SCR1<br>1 RPR1
**LTR**	22	

B

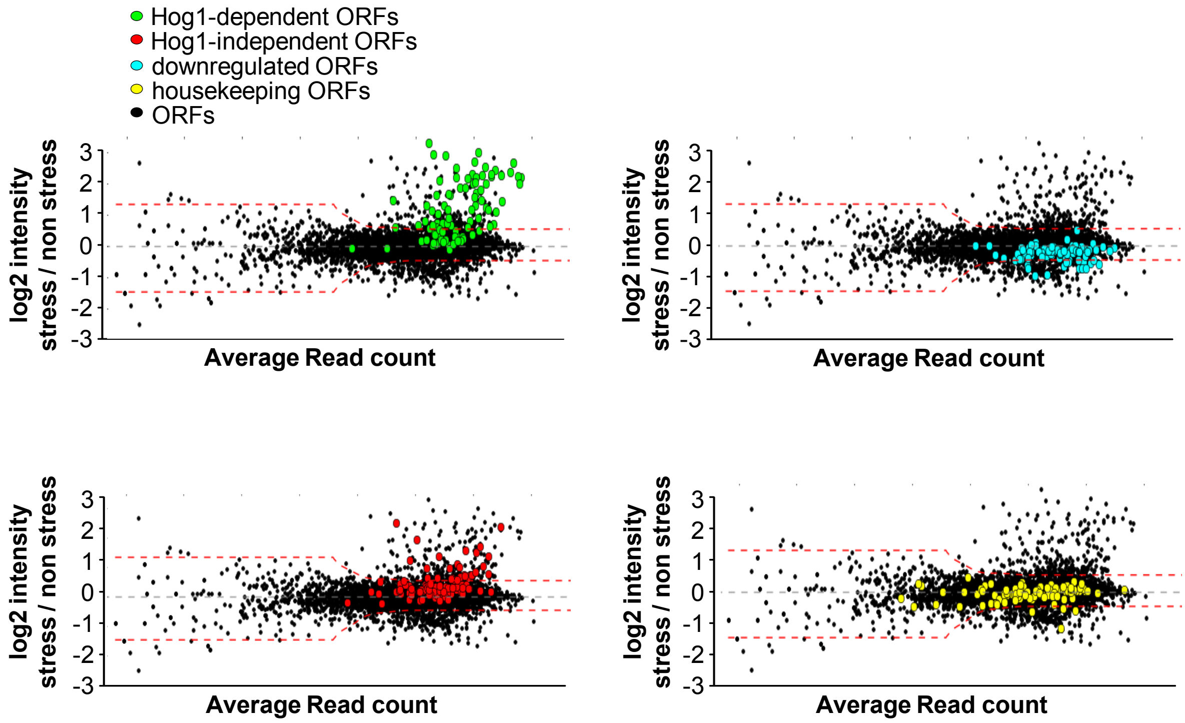

Fig S4 | A. Classification of Hog1 recruited genes or regions depending on their annotation in the SGD as RNA Pol II, RNA Pol III or Long Terminal Repeats (LTR). **B.** MA Plots representing Hog1 binding at the different sets of genes. A group of 100 Hog1-dependent genes are shown in green while 100 Hog1-independent genes are shown in red. In blue and yellow, sets of 100 genes that are downregulated and whose expression remains constant respectively as in Figure S3.

A

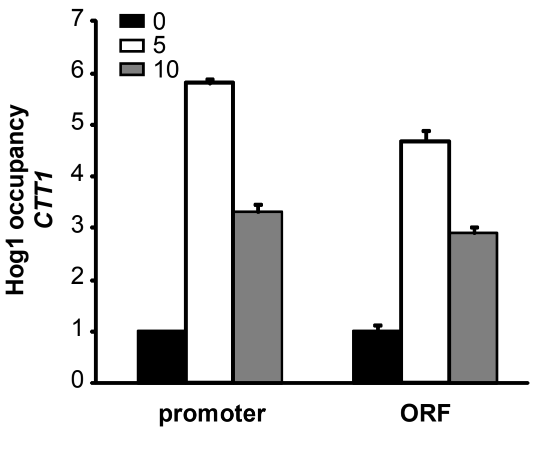

B

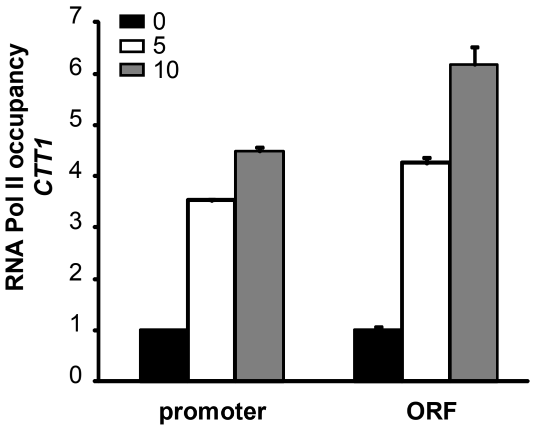

Fig S5 | Binding profile of Hog1 **(A)** and RNA Pol II **(B)** to *CTT1* osmo-responsive gene. Binding of Hog1 and RNA Pol II (Rpb1) validated by chromatin immunoprecipitation as in Figure 3D at the indicated regions of *CTT1* and times upon osmostress (0.4 M NaCl).

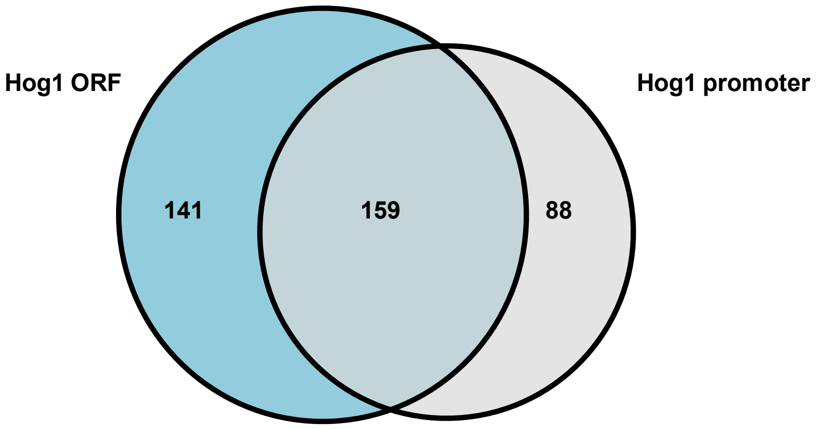

Fig S6 | Genome-wide distribution of Hog1. Venn diagram showing the number of genes in which the ORF and/or promoter (500 bp upstream of ATG) regions were occupied by Hog1 (z-score> 4).

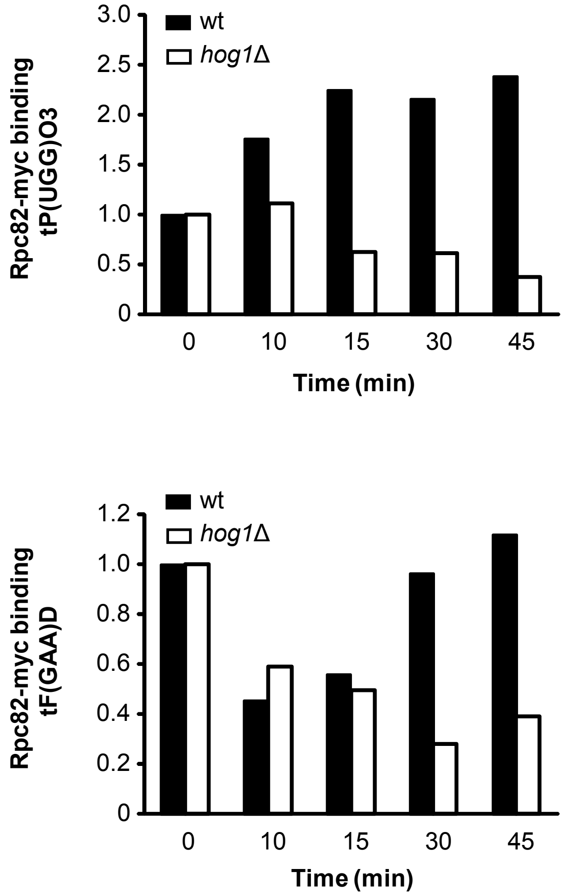

Fig S7 | Association of RNA PolII to tRNAs upon stress depends on *HOG1.* Association of specific subunit of RNA Pol III (Rpc82-myc) was assessed by ChIP upon osmostress (0.4M NaCl) at the indicated times and tRNA loci. Conventional PCR was performed and results are shown as fold induction of treated versus non-treated (time 0) and normalized against internal loading control (*TEL1*).

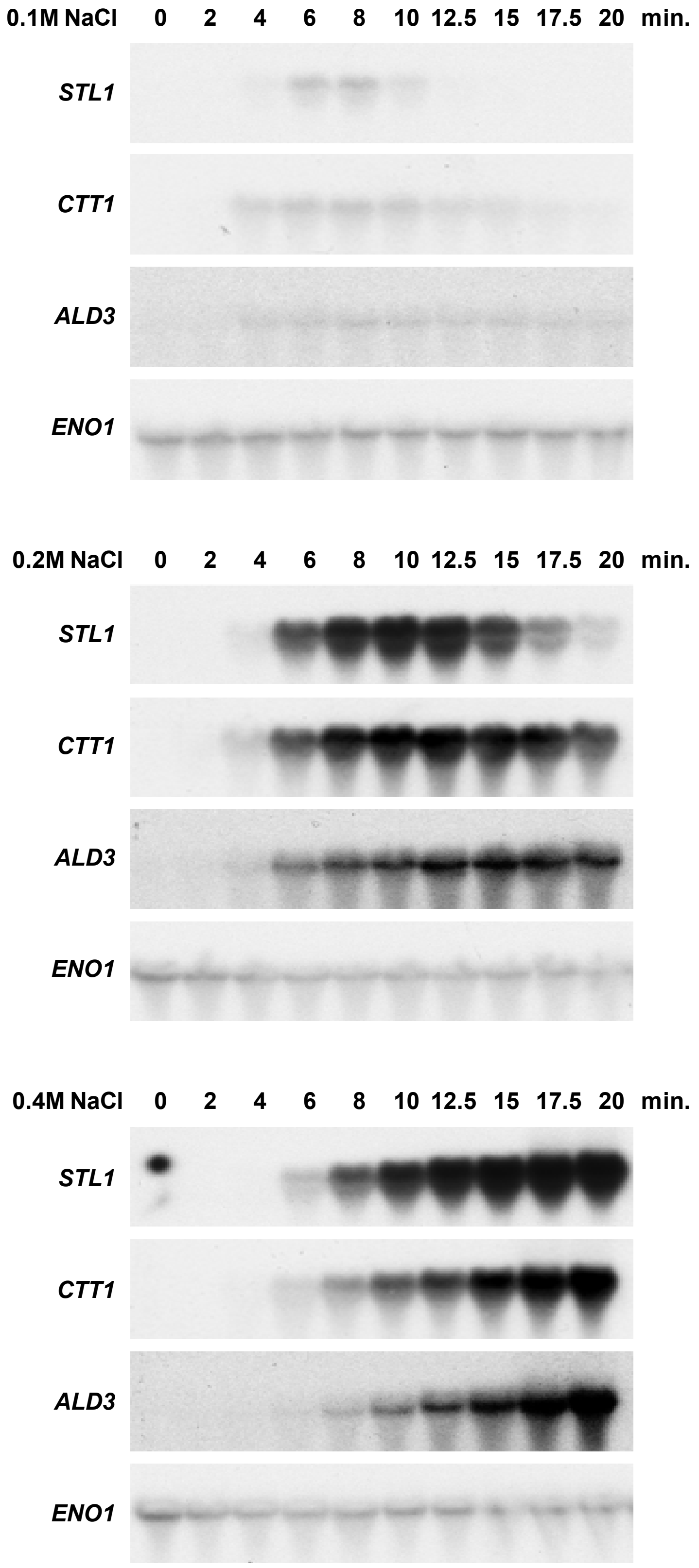

Fig S8 | Kinetics of dose response (0.1M, 0.2M and 0.4M NaCl) expression of different osmostress genes (*STL1*, *CTT1* and *ALD3*). Total RNA was extracted at the indicated times and concentrations. Expression of osmo-responsive genes was probed and normalized with *ENO1* as loading control.

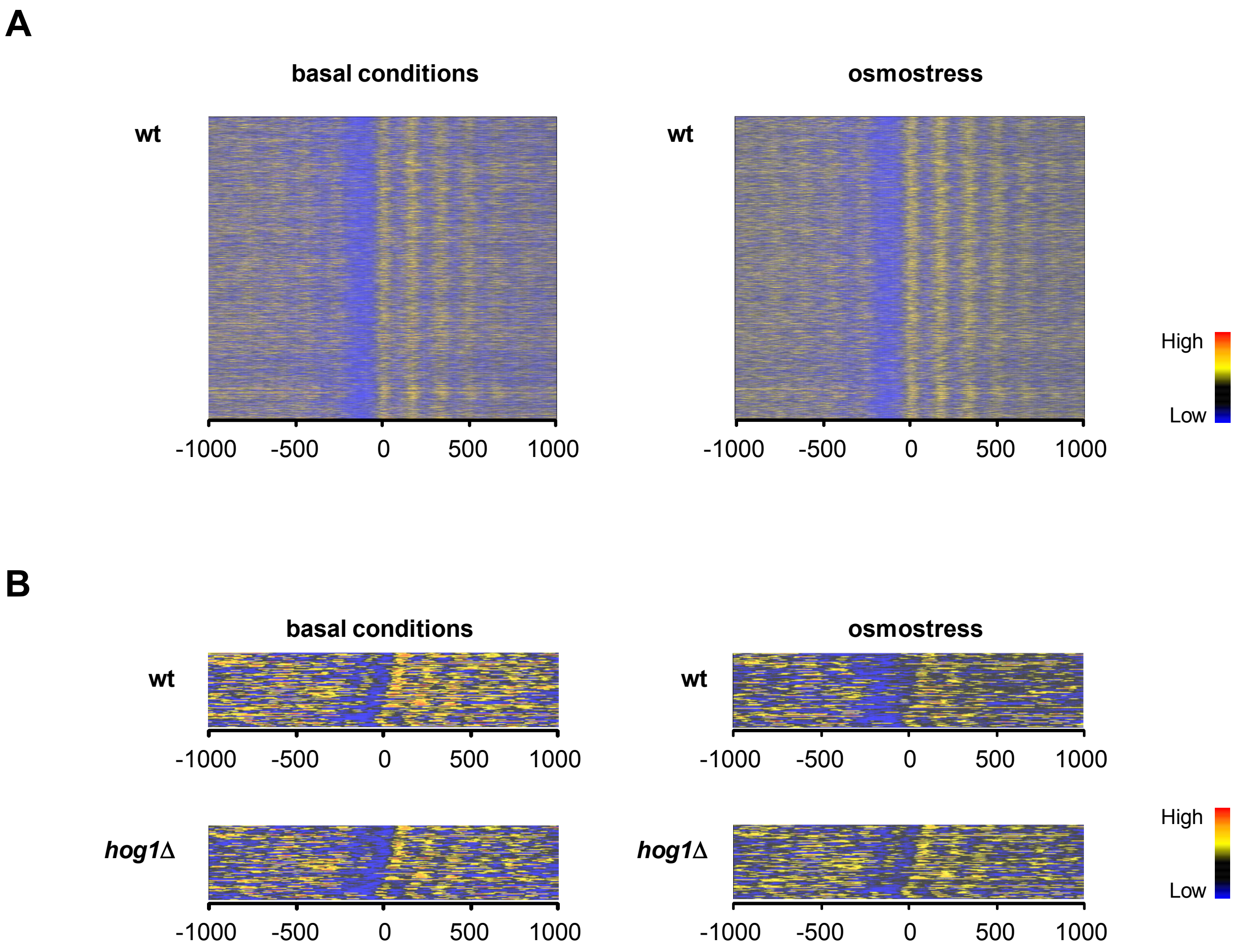

Fig S9 | Nucleosome occupancy maps determined by MNase-Seq genome-wide **(A)** and for Hog1-dependent genes (described in material and methods) in a *wild type* and *hog1* strains **(B)**. Coverage values of each position in the genome has been normalized, converted to reads per million (rpm) and represented in logarithmic scale . Genes (rows) are aligned by their TSS and sorted to maximize the correlation in 0 to 100 bp region. Blue, black, yellow and red indicate a low, medium, and high read density.
