# Supplementary material for: Hog1 bypasses stress-mediated down-regulation of transcription by RNA polymerase II redistribution and chromatin remodeling
Source: Genome Biol. 2012 Nov 18;13(11):R106. doi: 10.1186/gb-2012-13-11-r106 (PMC3580498; doi:10.1186/gb-2012-13-11-r106)
Supplement: Additional file 2 — Supplemetary Table 1. List of all the genes considered in the manuscript as Hog1-dependent (top 100 Hog1-depedent osmoresponsive genes), Hog1-independent (top 100 Hog1-independent osmoresponsive genes) and a list of all osmoresponsive genes from the microarray analysis (see the 'Gene expression studies' section in Materials and methods for the criteria used to define the genes present in the list). [file gb-2012-13-11-r106-S2.PDF]

**Table 1.** List of genes in Hog1 dependent, Hog1 independent and total osmoresponsive genes.

**Hog1 dependent genes**

| Systematic Name | Standard Name | Name |
|---|---|---|
| YBL064C | PRX1 | PeroxiRedoXin |
| YBR116C | | |
| YBR117C | TKL2 | TransKetoLase |
| YBR126C | TPS1 | Trehalose-6-Phosphate Synthase |
| YBR230C | OM14 | Outer Membrane Protein of 14 kDa |
| YCR010C | ADY2 | Accumulation of DYads |
| YEL011W | GLC3 | GLyCogen |
| YER054C | GIP2 | Glc7-Interacting Protein |
| YER062C | HOR2 | HyperOsmolarity-Responsive |
| YER067W | RGI1 | Respiratory Growth Induced |
| YER150W | SPI1 | Stationary Phase Induced |
| YHR022C | | |
| YHR033W | | |
| YHR087W | RTC3 | Restriction of Telomere Capping |
| YHR094C | HXT1 | HeXose Transporter |
| YHR096C | HXT5 | HeXose Transporter |
| YHR097C | | |
| YHR104W | GRE3 | Genes de Respuesta a Estres (stress responsive genes) |
| YHR139C | SPS100 | SPorulation Specific |
| YIL155C | GUT2 | Glycerol UTilization |
| YKL035W | UGP1 | UDP-glucose pyrophosphorylase |
| YKL091C | | |
| YKL093W | MBR1 | Mitochondrial Biogenesis Regulation |
| YKL096W | CWP1 | Cell Wall Protein |
| YKL151C | | |
| YKL163W | PIR3 | Protein containing Internal Repeats |
| YKR011C | | |
| YFL014W | HSP12 | Heat Shock Protein |
| YFR053C | HXK1 | HXK1 |
| YDL022W | GPD1 | Glycerol-3-Phosphate Dehydrogenase |
| YDL024C | DIA3 | Digs Into Agar |
| YDL110C | TMA17 | Translation Machinery Associated |
| YDL204W | RTN2 | ReTiculoN-like |
| YDL214C | PRR2 | Pheromone Response Regulator |
| YDL222C | FMP45 | Found in Mitochondrial Proteome |
| YDL223C | HBT1 | HuB1 Target |
| YDR070C | FMP16 | Found in Mitochondrial Proteome |
| YDR074W | TPS2 | Trehalose-6-phosphate PhoSphatase |
| YDR223W | CRF1 | Co-Repressor with FHL1 |
| YDR358W | GGA1 | Golgi-localized, Gamma-adaptin ear homology, Arf-binding protein |
| YDR516C | EMI2 | Early Meiotic Induction |
| YDR536W | STL1 | Sugar Transporter-Like protein |
| YGL157W | ARI1 | Aldehyde Reductase Intermediate, subclass of SDR |
| YGR043C | NQM1 | Non-Quiescent Mutant |
| YGR052W | FMP48 | Found in Mitochondrial Proteome |
| YGR066C | | |
| YGR067C | | |
| YGR070W | ROM1 | RhO1 Multicopy suppressor |
| YGR086C | PIL1 | Phosphorylation Inhibited by Long chain bases |
| YGR088W | CTT1 | CaTalase T |
| YGR144W | THI4 | THIamine metabolism |
| YGR161C | RTS3 | |
| YGR194C | XKS1 | XyluloKinaSe |
| YGR236C | SPG1 | Stationary Phase Gene |
| YGR243W | FMP43 | Found in Mitochondrial Proteome |
| YGR248W | SOL4 | Suppressor Of Los1-1 |
| YJL057C | IKS1 | |
| YJL107C | | |

| | | |
|---|---|---|
| YJL108C | PRM10 | Pheromone-Regulated Membrane protein |
| YJR036C | HUL4 | Hect Ubiquitin Ligase |
| YLL026W | HSP104 | Heat Shock Protein |
| YLR149C | | |
| YLR177W | | |
| YLR251W | SYM1 | Stress-inducible Yeast Mpv17 |
| YLR270W | DCS1 | DeCapping Scavenger |
| YLR312C | | |
| YLR327C | TMA10 | Translation Machinery Associated |
| YML100W | TSL1 | Trehalose Synthase Long chain |
| YML128C | MSC1 | Meiotic Sister-Chromatid recombination |
| YML131W | | |
| YMR081C | ISF1 | Increasing Suppression Factor |
| YMR105C | PGM2 | PhosphoGlucoMutase |
| YMR169C | ALD3 | ALdehyde Dehydrogenase |
| YMR173W | DDR48 | DNA Damage Responsive |
| YMR196W | | |
| YMR250W | GAD1 | GlutAmate Decarboxylase |
| YMR291W | TDA1 | Topoisomerase I Damage Affected |
| YNL036W | NCE103 | NonClassical Export |
| YNL194C | | |
| YNL195C | | |
| YNL274C | GOR1 | GlyOxylate Reductase |
| YNR002C | ATO2 | Ammonia (Ammonium) Transport Outward |
| YOL048C | RRT8 | Regulator of rDNA Transcription |
| YOL084W | PHM7 | PHosphate Metabolism |
| YOL151W | GRE2 | Genes de Respuesta a Estres (stress responsive genes) |
| YOR019W | | |
| YOR028C | CIN5 | Chromosome INstability |
| YOR052C | | |
| YOR134W | BAG7 | |
| YOR161C | PNS1 | pH Nine Sensitive |
| YOR173W | DCS2 | DeCapping Scavenger |
| YOR178C | GAC1 | Glycogen ACcumulation |
| YOR348C | PUT4 | Proline UTilization |
| YOR374W | ALD4 | ALdehyde Dehydrogenase |
| YPL061W | ALD6 | ALdehyde Dehydrogenase |
| YPL230W | USV1 | Up in StarVation |
| YPR149W | NCE102 | NonClassical Export |
| YPR160W | GPH1 | Glycogen PHosphorylase |
| YDR034W-B | | |
| YER053C-A | | |

**Hog1 independent genes**

| Systematic Name | Standard Name | Name |
|---|---|---|
| YAL028W | FRT2 | Functionally Related to TCP1 |
| YAL061W | BDH2 | |
| YBR066C | NRG2 | Negative Regulator of Glucose-controlled genes |
| YBR072W | HSP26 | Heat Shock Protein |
| YBR128C | ATG14 | AuTophaGy related |
| YBR165W | UBS1 | UBiquitin-conjugating enzyme Suppressor |
| YBR169C | SSE2 | |
| YBR183W | YPC1 | Yeast Phyto-Ceramidase |
| YBR214W | SDS24 | homolog of S. pombe SDS23 |
| YBR284W | | |
| YCL040W | GLK1 | GLucoKinase |
| YCL042W | | |
| YEL039C | CYC7 | CYtochrome C |
| YER037W | PHM8 | PHosphate Metabolism |
| YER079W | | |
| YER103W | SSA4 | Stress-Seventy subfamily A |
| YER175C | TMT1 | Trans-aconitate MethylTransferase |
| YHL021C | AIM17 | Altered Inheritance rate of Mitochondria |
| YHL024W | RIM4 | Regulator of IME2 |
| YHR137W | ARO9 | AROmatic amino acid requiring |
| YIL055C | | |
| YIL056W | VHR1 | VHt1 Regulator |
| YIL077C | | |
| YIL107C | PFK26 | 6-PhosphoFructo-2-Kinase |
| YIL113W | SDP1 | Stress-inducible Dual specificity Phosphatase |
| YIL136W | OM45 | Outer Membrane |
| YIR014W | | |
| YIR017C | MET28 | METhionine requiring |
| YKL023W | | |
| YKL142W | MRP8 | Mitochondrial Ribosomal Protein |
| YFL016C | MDJ1 | Mitochondrial DnaJ |
| YFR015C | GSY1 | Glycogen SYnthase |
| YFR017C | IGD1 | Inhibitor of Glycogen Debranching |
| YDL010W | GRX6 | GlutaRedoXin |
| YDL020C | RPN4 | Regulatory Particle Non-ATPase |
| YDL124W | | |
| YDR001C | NTH1 | Neutral TreHalase |
| YDR171W | HSP42 | Heat Shock Protein |
| YDR185C | UPS3 | UnProceSsed |
| YDR273W | DON1 | DONut |
| YDR277C | MTH1 | MSN Three Homolog |
| YDR380W | ARO10 | AROmatic amino acid requiring |
| YDR406W | PDR15 | Pleiotropic Drug Resistance |
| YDR512C | EMI1 | Early Meiotic Induction |
| YDR523C | SPS1 | SPorulation Specific |
| YDR540C | IRC4 | Increased Recombination Centers |
| YGL010W | | |
| YGL036W | | |
| YGL146C | RRT6 | Regulator of rDNA Transcription |
| YGL158W | RCK1 | Radiation sensitivity Complementing Kinase |
| YGL166W | CUP2 | |
| YGR008C | STF2 | STabilizing Factor |
| YGR131W | FHN1 | Functional Homologue of Nce102 |
| YGR142W | BTN2 | BaTteN disease |
| YJL144W | | |
| YJL153C | INO1 | INOsitol requiring |
| YJL155C | FBP26 | Fructose BisPhosphatase |
| YJL161W | FMP33 | Found in Mitochondrial Proteome |
| YJL163C | | |
| YJR059W | PTK2 | Putative serine/Threonine protein Kinase |

| | | |
|---|---|---|
| YJR149W | | |
| YLL039C | UBI4 | Ubiquitin |
| YLR081W | GAL2 | GALactose metabolism |
| YLR099C | ICT1 | Increased Copper Tolerance |
| YLR152C | | |
| YLR164W | SHH4 | SDH4 Homolog |
| YLR174W | IDP2 | Isocitrate Dehydrogenase, NADP-specific |
| YLR225C | | |
| YLR267W | BOP2 | Bypass Of Pam1 |
| YLR345W | | |
| YLR350W | ORM2 | |
| YLR446W | | |
| YMR009W | ADI1 | Acireductone DIoxygenase |
| YMR031C | EIS1 | EISosome |
| YMR034C | | |
| YMR084W | | |
| YMR206W | | |
| YMR251W-A | HOR7 | HyperOsmolarity-Responsive |
| YNL011C | | |
| YNL077W | APJ1 | Anti-Prion DnaJ |
| YNL159C | ASI2 | Amino acid Sensor-Independent |
| YNL160W | YGP1 | Yeast GlycoProtein |
| YNR014W | | |
| YOL014W | | |
| YOR003W | YSP3 | Yeast Subtilisin-like Protease III |
| YOR049C | RSB1 | Resistance to Sphingoid long-chain Base |
| YOR186W | | |
| YOR220W | RCN2 | Regulator of CalciNeurin |
| YOR273C | TPO4 | Transporter of POlyamines |
| YOR289W | | |
| YPL014W | | |
| YPL247C | | |
| YPR026W | ATH1 | Acid TreHalase |
| YPR158W | CUR1 | Curing of [URe3] |
| YDL130W-A | STF1 | STabilizing Factor |
| YBL029C-A | | |
| YPR036W-A | | |
| YOR020W-A | | |
| YLR361C-A | | |
| YGR161W-C | | |

**Total osmoresponsive genes**

| Systematic Name | Standard Name | Name |
| --- | --- | --- |
| YAL028W | FRT2 | Functionally Related to TCP1 |
| YAL032C | PRP45 | Pre-mRNA Processing |
| YAL039C | CYC3 | CYtochrome C |
| YAL040C | CLN3 | CycLiN |
| YAL060W | BDH1 | Butanediol DeHydrogenase |
| YAL061W | BDH2 | |
| YAR020C | PAU7 | seriPAUperin family |
| YBL064C | PRX1 | PeroxiRedoXin |
| YBL078C | ATG8 | AuTophaGy related |
| YBL086C | | |
| YBR005W | RCR1 | Resistance to Congo Red |
| YBR006W | UGA2 | Utilization of GAba |
| YBR014C | GRX7 | GlutaRedoXin |
| YBR018C | GAL7 | GALactose metabolism |
| YBR021W | FUR4 | 5-FlUoRouridine sensitivity |
| YBR024W | SCO2 | Suppressor of Cytochrome Oxidase deficiency |
| YBR031W | RPL4A | Ribosomal Protein of the Large subunit |
| YBR037C | SCO1 | Suppressor of Cytochrome Oxidase deficiency |
| YBR045C | GIP1 | Glc7-Interacting Protein |
| YBR050C | REG2 | REsistance to Glucose repression |
| YBR051W | | |
| YBR052C | RFS1 | Rad55 (Fifty-five) Suppressor |
| YBR054W | YRO2 | |
| YBR056W | | |
| YBR066C | NRG2 | Negative Regulator of Glucose-controlled genes |
| YBR072W | HSP26 | Heat Shock Protein |
| YBR078W | ECM33 | ExtraCellular Mutant |
| YBR099C | | |
| YBR114W | RAD16 | RADiation sensitive |
| YBR116C | | |
| YBR117C | TKL2 | TransKetoLase |
| YBR118W | TEF2 | Translation Elongation Factor |
| YBR119W | MUD1 | Mutant U1 Die |
| YBR126C | TPS1 | Trehalose-6-Phosphate Synthase |
| YBR132C | AGP2 | high-Affinity Glutamine Permease |
| YBR139W | | |
| YBR148W | YSW1 | |
| YBR149W | ARA1 | D-ARAbinose dehydrogenase |
| YBR160W | CDC28 | Cell Division Cycle |
| YBR161W | CSH1 | CSG1/SUR1 Homolog |
| YBR169C | SSE2 | |
| YBR177C | EHT1 | Ethanol Hexanoyl Transferase |
| YBR182C | SMP1 | Second MEF2-like Protein 1 |
| YBR183W | YPC1 | Yeast Phyto-Ceramidase |
| YBR196C | PGI1 | PhosphoGlucoIsomerase |
| YBR203W | COS111 | Ciclopirox Olamine Sensitive |
| YBR212W | NGR1 | Negative Growth Regulatory protein |
| YBR214W | SDS24 | homolog of S. pombe SDS23 |
| YBR230C | OM14 | Outer Membrane Protein of 14 kDa |
| YBR237W | PRP5 | Pre-mRNA Processing |

| | | |
|---|---|---|
| YBR269C | FMP21 | Found in Mitochondrial Proteome |
| YBR284W | | |
| YBR286W | APE3 | AminoPEptidase |
| YBR287W | | |
| YBR296C | PHO89 | PHOsphate metabolism |
| YBR298C | MAL31 | MALtose fermentation |
| YCL027W | FUS1 | cell FUSion |
| YCL039W | GID7 | Glucose Induced Degradation deficient |
| YCL040W | GLK1 | GLucoKinase |
| YCL046W | | |
| YCL049C | | |
| YCL026C-A | FRM2 | Fatty acid Repression Mutant |
| YCR010C | ADY2 | Accumulation of DYads |
| YCR012W | PGK1 | 3-PhosphoGlycerate Kinase |
| YCR020C | PET18 | PETite colonies |
| YCR021C | HSP30 | Heat Shock Protein |
| YCR022C | | |
| YCR030C | SYP1 | Suppressor of Yeast Profilin deletion |
| YCR061W | | |
| YCR073W-A | SOL2 | Suppressor Of Los1-1 |
| YEL011W | GLC3 | GLyCogen |
| YEL033W | MTC7 | Maintenance of Telomere Capping |
| YEL039C | CYC7 | CYtochrome C |
| YEL060C | PRB1 | PRoteinase B |
| YER020W | GPA2 | G Protein Alpha subunit |
| YER035W | EDC2 | Enhancer of mRNA DeCapping |
| YER037W | PHM8 | PHosphate Metabolism |
| YER039C | HVG1 | Homologous to VRG4 |
| YER053C | PIC2 | PI Carrier |
| YER054C | GIP2 | Glc7-Interacting Protein |
| YER062C | HOR2 | HyperOsmolarity-Responsive |
| YER063W | THO1 | suppressor of the Transcriptional defect of Hpr1 by Overexpression |
| YER065C | ICL1 | IsoCitrate Lyase |
| YER066W | RRT13 | Regulator of rDNA Transcription |
| YER067W | RGI1 | Respiratory Growth Induced |
| YER079W | | |
| YER081W | SER3 | SERine requiring |
| YER095W | RAD51 | RADiation sensitive |
| YER096W | SHC1 | Sporulation-specific Homolog of CSD4 |
| YER097W | | |
| YER103W | SSA4 | Stress-Seventy subfamily A |
| YER150W | SPI1 | Stationary Phase Induced |
| YER188W | | |
| YHL002W | HSE1 | Has Symptoms of class E mutants; resembles Hbp, Stam and East |
| YHL016C | DUR3 | Degradation of URea |
| YHL021C | AIM17 | Altered Inheritance rate of Mitochondria |
| YHL024W | RIM4 | Regulator of IME2 |
| YHL044W | | |
| YHR016C | YSC84 | |
| YHR022C | | |
| YHR030C | SLT2 | Suppression at Low Temperature |
| YHR031C | RRM3 | rDNA Recombination Mutation |
| YHR033W | | |

| | | |
|---|---|---|
| YHR049W | FSH1 | Family of Serine Hydrolases |
| YHR075C | PPE1 | Phosphoprotein Phosphatase methylEsterase |
| YHR087W | RTC3 | Restriction of Telomere Capping |
| YHR092C | HXT4 | HeXose Transporter |
| YHR094C | HXT1 | HeXose Transporter |
| YHR095W | | |
| YHR096C | HXT5 | HeXose Transporter |
| YHR097C | | |
| YHR104W | GRE3 | Genes de Respuesta a Estres (stress responsive genes) |
| YHR124W | NDT80 | Non-DiTyrosine |
| YHR137W | ARO9 | AROmatic amino acid requiring |
| YHR138C | | |
| YHR139C | SPS100 | SPorulation Specific |
| YHR161C | YAP1801 | Yeast Assembly Polypeptide |
| YHR171W | ATG7 | AuTophaGy related |
| YHR174W | ENO2 | ENO2 |
| YHR195W | NVJ1 | Nucleus-Vacuole Junction |
| YIL017C | VID28 | Vacuolar Import and Degradation |
| YIL018W | RPL2B | Ribosomal Protein of the Large subunit |
| YIL024C | | |
| YIL033C | BCY1 | Bypass of CYclase mutations |
| YIL036W | CST6 | Chromosome STability |
| YIL042C | PKP1 | Protein Kinase of PDH |
| YIL045W | PIG2 | Protein Interacting with Gsy2p |
| YIL053W | RHR2 | Related to HoR2 |
| YIL054W | | |
| YIL055C | | |
| YIL056W | VHR1 | VHt1 Regulator |
| YIL077C | | |
| YIL099W | SGA1 | Sporulation-specific GlycoAmylase |
| YIL100W | | |
| YIL101C | XBP1 | XhoI site-Binding Protein |
| YIL105C | SLM1 | Synthetic Lethal with Mss4 |
| YIL107C | PFK26 | 6-PhosphoFructo-2-Kinase |
| YIL108W | | |
| YIL111W | COX5B | Cytochrome c OXidase |
| YIL113W | SDP1 | Stress-inducible Dual specificity Phosphatase |
| YIL136W | OM45 | Outer Membrane |
| YIL155C | GUT2 | Glycerol UTilization |
| YIL160C | POT1 | Peroxisomal Oxoacyl Thiolase |
| YIR007W | | |
| YIR013C | GAT4 | |
| YIR014W | | |
| YIR016W | | |
| YIR017C | MET28 | METhionine requiring |
| YIR032C | DAL3 | Degradation of Allantoin |
| YKL007W | CAP1 | CAPping |
| YKL023W | | |
| YKL035W | UGP1 | UDP-glucose pyrophosphorylase |
| YKL051W | SFK1 | Suppressor of Four Kinase |
| YKL053W | | |
| YKL059C | MPE1 | Mutant PCF11 Extragenic suppressor |
| YKL060C | FBA1 | FBA1 |

| | | |
|---|---|---|
| YKL065C | YET1 | Yeast Endoplasmic reticulum Transmembrane protein |
| YKL066W | | |
| YKL091C | | |
| YKL093W | MBR1 | Mitochondrial Biogenesis Regulation |
| YKL096W | CWP1 | Cell Wall Protein |
| YKL097C | | |
| YKL103C | APE1 | AminoPeptidase |
| YKL123W | | |
| YKL124W | SSH4 | Suppressor of SHr3 deletion |
| YKL133C | | |
| YKL142W | MRP8 | Mitochondrial Ribosomal Protein |
| YKL146W | AVT3 | Amino acid Vacuolar Transport |
| YKL148C | SDH1 | Succinate DeHydrogenase |
| YKL150W | MCR1 | Mitochondrial NADH-Cytochrome b5 Reductase |
| YKL151C | | |
| YKL152C | GPM1 | Glycerate PhosphoMutase |
| YKL163W | PIR3 | Protein containing Internal Repeats |
| YKL169C | | |
| YKL193C | SDS22 | homolog of S. pombe SDS22 |
| YKL217W | JEN1 | |
| YKR005C | | |
| YKR011C | | |
| YKR035C | OPI8 | OverProducer of Inositol |
| YKR039W | GAP1 | General Amino acid Permease |
| YKR046C | PET10 | PETite colonies |
| YKR049C | FMP46 | Found in Mitochondrial Proteome |
| YKR052C | MRS4 | Mitochondrial RNA Splicing |
| YKR058W | GLG1 | Glycogenin-Like Gene |
| YKR067W | GPT2 | Glycerol-3-Phosphate acylTransferase |
| YKR089C | TGL4 | TriacylGlycerol Lipase |
| YKR091W | SRL3 | Suppressor of rad53 Lethality |
| YFL039C | ACT1 | ACTin |
| YFL031W | HAC1 | Homologous to Atf/Creb1 |
| YFL030W | AGX1 | Alanine:Glyoxylate aminotrans(X)ferase |
| YFL021W | GAT1 | |
| YFL016C | MDJ1 | Mitochondrial DnaJ |
| YFL014W | HSP12 | Heat Shock Protein |
| YFR003C | YPI1 | Yeast Phosphatase Inhibitor |
| YFR014C | CMK1 | CalModulin dependent protein Kinase |
| YFR015C | GSY1 | Glycogen SYnthase |
| YFR017C | IGD1 | Inhibitor of Glycogen Debranching |
| YFR022W | ROG3 | Revertant Of Glycogen synthase kinase mutation |
| YFR044C | DUG1 | Deficient in Utilization of Glutathione |
| YFR053C | HXK1 | HXK1 |
| YFR031C-A | RPL2A | Ribosomal Protein of the Large subunit |
| YAL034C | FUN19 | Function Unknown Now |
| YDL010W | GRX6 | GlutaRedoXin |
| YDL013W | SLX5 | Synthetic Lethal of unknown [X] function |
| YDL019C | OSH2 | OxySterol binding protein Homolog |
| YDL020C | RPN4 | Regulatory Particle Non-ATPase |
| YDL021W | GPM2 | Glycerate PhosphoMutase |
| YDL022W | GPD1 | Glycerol-3-Phosphate Dehydrogenase |
| YDL023C | | |

| | | |
|---|---|---|
| YDL024C | DIA3 | Digs Into Agar |
| YDL025C | RTK1 | Ribosome biogenesis and TRNA synthetase-associated Kinase |
| YDL026W | | |
| YDL048C | STP4 | protein with similarity to Stp1p |
| YDL079C | MRK1 | Mds1p Related Kinase |
| YDL110C | TMA17 | Translation Machinery Associated |
| YDL123W | SNA4 | Sensitivity to NA+ |
| YDL124W | | |
| YDL125C | HNT1 | Histidine triad NucleoTide-binding |
| YDL128W | VCX1 | VaCuolar H+/Ca2+ eXchanger |
| YDL161W | ENT1 | Epsin N-Terminal homology |
| YDL169C | UGX2 | Unidentified Gene X |
| YDL199C | | |
| YDL204W | RTN2 | ReTiculoN-like |
| YDL206W | | |
| YDL214C | PRR2 | Pheromone Response Regulator |
| YDL222C | FMP45 | Found in Mitochondrial Proteome |
| YDL223C | HBT1 | HuB1 Target |
| YDL230W | PTP1 | Protein Tyrosine Phosphatase |
| YDL247W | MPH2 | Maltose Permease Homolog |
| YDR001C | NTH1 | Neutral TreHalase |
| YDR003W | RCR2 | Resistance to Congo Red |
| YDR027C | VPS54 | Vacuolar Protein Sorting |
| YDR039C | ENA2 | Exitus NAtru (Latin, "exit sodium") |
| YDR040C | ENA1 | Exitus NAtru (Latin, "exit sodium") |
| YDR042C | | |
| YDR043C | NRG1 | Negative Regulator of Glucose-repressed genes |
| YDR050C | TPI1 | Triose-Phosphate Isomerase |
| YDR058C | TGL2 | TriacylGlycerol Lipase |
| YDR063W | AIM7 | Altered Inheritance rate of Mitochondria |
| YDR069C | DOA4 | Degradation Of Alpha |
| YDR070C | FMP16 | Found in Mitochondrial Proteome |
| YDR072C | IPT1 | InositolPhosphoTransferase |
| YDR073W | SNF11 | Sucrose NonFermenting |
| YDR074W | TPS2 | Trehalose-6-phosphate PhoSphatase |
| YDR085C | AFR1 | Alpha-Factor Receptor regulator |
| YDR088C | SLU7 | Synergistic Lethal with U5 snRNA |
| YDR096W | GIS1 | GIg1-2 Suppressor |
| YDR122W | KIN1 | KINase |
| YDR133C | | |
| YDR153C | ENT5 | Epsin N-Terminal homology |
| YDR171W | HSP42 | Heat Shock Protein |
| YDR173C | ARG82 | ARGinine requiring |
| YDR185C | UPS3 | UnProceSsed |
| YDR216W | ADR1 | Alcohol Dehydrogenase Regulator |
| YDR223W | CRF1 | Co-Repressor with FHL1 |
| YDR229W | IVY1 | Interacting with Vps33p and Ypt7p |
| YDR231C | COX20 | Cytochrome c OXidase |
| YDR247W | VHS1 | Viable in a Hal3 Sit4 background |
| YDR258C | HSP78 | Heat Shock Protein |
| YDR273W | DON1 | DONut |
| YDR305C | HNT2 | Histidine triad NucleoTide-binding |
| YDR330W | UBX5 | UBiquitin regulatory X |

| | | |
|---|---|---|
| YDR342C | HXT7 | HeXose Transporter |
| YDR343C | HXT6 | HeXose Transporter |
| YDR358W | GGA1 | Golgi-localized, Gamma-adaptin ear homology, Arf-binding protein |
| YDR380W | ARO10 | AROmatic amino acid requiring |
| YDR385W | EFT2 | Elongation Factor Two |
| YDR387C | | |
| YDR391C | | |
| YDR392W | SPT3 | SuPpressor of Ty's |
| YDR402C | DIT2 | DITyrosine |
| YDR406W | PDR15 | Pleiotropic Drug Resistance |
| YDR408C | ADE8 | ADEnine requiring |
| YDR476C | | |
| YDR494W | RSM28 | Ribosomal Small subunit of Mitochondria |
| YDR501W | PLM2 | PLasmid Maintenance |
| YDR508C | GNP1 | GlutamiNe Permease |
| YDR509W | | |
| YDR513W | GRX2 | GlutaRedoXin |
| YDR516C | EMI2 | Early Meiotic Induction |
| YDR523C | SPS1 | SPorulation Specific |
| YDR529C | QCR7 | ubiQuinol-cytochrome C oxidoReductase |
| YDR530C | APA2 | AP4A phosphorylase |
| YDR533C | HSP31 | Heat-Shock Protein |
| YDR534C | FIT1 | Facilitator of Iron Transport |
| YDR536W | STL1 | Sugar Transporter-Like protein |
| YDR543C | | |
| YER093C-A | AIM11 | Altered Inheritance rate of Mitochondria |
| YGL006W | PMC1 | Plasma Membrane Calcium |
| YGL007W | BRP1 | |
| YGL010W | | |
| YGL012W | ERG4 | ERGosterol biosynthesis |
| YGL024W | | |
| YGL036W | | |
| YGL037C | PNC1 | Pyrazinamidase and NiCotinamidase |
| YGL045W | RIM8 | Regulator of IME2 |
| YGL051W | MST27 | Multicopy suppressor of Sec Twenty one |
| YGL052W | | |
| YGL053W | PRM8 | Pheromone-Regulated Membrane protein |
| YGL104C | VPS73 | Vacuolar Protein Sorting |
| YGL123W | RPS2 | Ribosomal Protein of the Small subunit |
| YGL127C | SOH1 | Suppressor Of Hpr1 |
| YGL156W | AMS1 | Alpha-MannoSidase |
| YGL157W | ARI1 | Aldehyde Reductase Intermediate, subclass of SDR |
| YGL158W | RCK1 | Radiation sensitivity Complementing Kinase |
| YGL166W | CUP2 | |
| YGL179C | TOS3 | Target Of Sbf |
| YGL189C | RPS26A | Ribosomal Protein of the Small subunit |
| YGL222C | EDC1 | Enhancer of mRNA DeCapping |
| YGL225W | VRG4 | Vandate Resistance Glycosylation |
| YGL229C | SAP4 | Sit4 Associated Protein |
| YGL237C | HAP2 | Heme Activator Protein |
| YGL250W | RMR1 | Reduced Meiotic Recombination |
| YGL253W | HXK2 | HXK2 |
| YGL255W | ZRT1 | Zinc-Regulated Transporter |

| | | |
|---|---|---|
| YGR008C | STF2 | STabilizing Factor |
| YGR018C | | |
| YGR023W | MTL1 | Mid-Two Like |
| YGR043C | NQM1 | Non-Quiescent Mutant |
| YGR045C | | |
| YGR046W | TAM41 | Translocator Assembly and Maintenance 41 |
| YGR050C | | |
| YGR052W | FMP48 | Found in Mitochondrial Proteome |
| YGR053C | | |
| YGR066C | | |
| YGR067C | | |
| YGR069W | | |
| YGR070W | ROM1 | RhO1 Multicopy suppressor |
| YGR086C | PIL1 | Phosphorylation Inhibited by Long chain bases |
| YGR087C | PDC6 | Pyruvate DeCarboxylase |
| YGR088W | CTT1 | CaTalase T |
| YGR121C | MEP1 | |
| YGR122W | | |
| YGR127W | | |
| YGR130C | | |
| YGR131W | FHN1 | Functional Homologue of Nce102 |
| YGR142W | BTN2 | BaTteN disease |
| YGR144W | THI4 | THIamine metabolism |
| YGR149W | | |
| YGR160W | | |
| YGR161C | RTS3 | |
| YGR182C | | |
| YGR190C | | |
| YGR192C | TDH3 | Triose-phosphate DeHydrogenase |
| YGR194C | XKS1 | XyluloKinaSe |
| YGR205W | TDA10 | Topoisomerase I Damage Affected |
| YGR222W | PET54 | PETite colonies |
| YGR230W | BNS1 | Bypasses Need for Spo12p |
| YGR236C | SPG1 | Stationary Phase Gene |
| YGR237C | | |
| YGR241C | YAP1802 | Yeast Assembly Polypeptide |
| YGR243W | FMP43 | Found in Mitochondrial Proteome |
| YGR248W | SOL4 | Suppressor Of Los1-1 |
| YGR249W | MGA1 | |
| YGR254W | ENO1 | ENO1 |
| YGR256W | GND2 | |
| YGR282C | BGL2 | Beta-GLucanase |
| YGR289C | MAL11 | MALtose fermentation |
| YGR291C | | |
| YJL048C | UBX6 | UBiquitin regulatory X |
| YJL057C | IKS1 | |
| YJL064W | | |
| YJL066C | MPM1 | Mitochondrial Peculiar Membrane protein |
| YJL070C | | |
| YJL106W | IME2 | Inducer of MEiosis |
| YJL107C | | |
| YJL108C | PRM10 | Pheromone-Regulated Membrane protein |
| YJL132W | | |

| | | |
|---|---|---|
| YJL141C | YAK1 | Yet Another Kinase |
| YJL142C | IRC9 | Increased Recombination Centers |
| YJL144W | | |
| YJL153C | INO1 | INOsitol requiring |
| YJL155C | FBP26 | Fructose BisPhosphatase |
| YJL161W | FMP33 | Found in Mitochondrial Proteome |
| YJL163C | | |
| YJL164C | TPK1 | Takashi's Protein Kinase |
| YJL165C | HAL5 | HALotolerance |
| YJL171C | | |
| YJL188C | BUD19 | BUD site selection |
| YJL196C | ELO1 | ELOngation defective |
| YJR008W | MHO1 | Memo HOmolog |
| YJR019C | TES1 | ThioESterase |
| YJR036C | HUL4 | Hect Ubiquitin Ligase |
| YJR059W | PTK2 | Putative serine/Threonine protein Kinase |
| YJR079W | | |
| YJR086W | STE18 | STErile |
| YJR087W | | |
| YJR104C | SOD1 | SuperOxide Dismutase |
| YJR115W | | |
| YJR120W | | |
| YJR123W | RPS5 | Ribosomal Protein of the Small subunit |
| YJR149W | | |
| YJR160C | MPH3 | Maltose Permease Homolog |
| YLL019C | KNS1 | Kinase Next to SPA2 |
| YLL020C | | |
| YLL023C | POM33 | POre Membrane, 33 kDa |
| YLL025W | PAU17 | seriPAUperin |
| YLL026W | HSP104 | Heat Shock Protein |
| YLL027W | ISA1 | Iron Sulfur Assembly |
| YLL028W | TPO1 | Transporter of POlyamines |
| YLL039C | UBI4 | Ubiquitin |
| YLL041C | SDH2 | Succinate DeHydrogenase |
| YLR006C | SSK1 | Suppressor of Sensor Kinase |
| YLR029C | RPL15A | Ribosomal Protein of the Large subunit |
| YLR031W | | |
| YLR042C | | |
| YLR081W | GAL2 | GALactose metabolism |
| YLR099C | ICT1 | Increased Copper Tolerance |
| YLR108C | | |
| YLR111W | | |
| YLR112W | | |
| YLR120C | YPS1 | YaPSin |
| YLR121C | YPS3 | YaPSin |
| YLR149C | | |
| YLR151C | PCD1 | Peroxisomal Coenzyme A Diphosphatase |
| YLR152C | | |
| YLR155C | ASP3-1 | ASParaginase |
| YLR157C | ASP3-2 | ASParaginase |
| YLR158C | ASP3-3 | ASParaginase |
| YLR160C | ASP3-4 | ASParaginase |
| YLR164W | SHH4 | SDH4 Homolog |

| | | |
|---|---|---|
| YLR165C | PUS5 | PseudoUridine Synthase |
| YLR167W | RPS31 | Ribosomal Protein of the Small subunit |
| YLR171W | | |
| YLR174W | IDP2 | Isocitrate Dehydrogenase, NADP-specific |
| YLR176C | RFX1 | |
| YLR177W | | |
| YLR178C | TFS1 | cdc25 (Twenty-Five) Suppressor |
| YLR194C | | |
| YLR219W | MSC3 | Meiotic Sister-Chromatid recombination |
| YLR225C | | |
| YLR251W | SYM1 | Stress-inducible Yeast Mpv17 |
| YLR252W | | |
| YLR254C | NDL1 | NuDeL homolog |
| YLR256W | HAP1 | Heme Activator Protein |
| YLR257W | | |
| YLR258W | GSY2 | Glycogen SYnthase |
| YLR267W | BOP2 | Bypass Of Pam1 |
| YLR270W | DCS1 | DeCapping Scavenger |
| YLR284C | ECI1 | Enoyl-CoA Isomerase |
| YLR311C | | |
| YLR312C | | |
| YLR327C | TMA10 | Translation Machinery Associated |
| YLR329W | REC102 | RECombination |
| YLR340W | RPP0 | Ribosomal Protein P0 |
| YLR343W | GAS2 | Glycophospholipid-Anchored Surface protein |
| YLR345W | | |
| YLR350W | ORM2 | |
| YLR414C | PUN1 | Plasma membrane protein Upregulated during Nitrogen stress |
| YLR417W | VPS36 | Vacuolar Protein Sorting |
| YLR438W | CAR2 | Catabolism of ARginine |
| YLR446W | | |
| YML004C | GLO1 | GLyOxalase |
| YML030W | RCF1 | Respiratory superComplex Factor |
| YML053C | | |
| YML054C | CYB2 | CYtochrome B |
| YML070W | DAK1 | DihydroxyAcetone Kinase |
| YML100W | TSL1 | Trehalose Synthase Long chain |
| YML120C | NDI1 | NADH Dehydrogenase Internal |
| YML128C | MSC1 | Meiotic Sister-Chromatid recombination |
| YML131W | | |
| YMR009W | ADI1 | Acireductone DIoxygenase |
| YMR017W | SPO20 | SPOrulation |
| YMR031C | EIS1 | EISosome |
| YMR032W | HOF1 | Homolog Of cdc Fifteen |
| YMR034C | | |
| YMR037C | MSN2 | Multicopy suppressor of SNF1 mutation |
| YMR039C | SUB1 | SUppressor of TFIIB mutations |
| YMR040W | YET2 | Yeast Endoplasmic reticulum Transmembrane protein |
| YMR043W | MCM1 | MiniChromosome Maintenance |
| YMR052C-A | | |
| YMR053C | STB2 | Sin Three Binding protein |
| YMR068W | AVO2 | Adheres VOraciously (to TOR2) |
| YMR069W | NAT4 | N-AcetylTransferase |

| | | |
|---|---|---|
| YMR070W | MOT3 | Modifier of Transcription |
| YMR081C | ISF1 | Increasing Suppression Factor |
| YMR084W | | |
| YMR085W | | |
| YMR087W | | |
| YMR090W | | |
| YMR102C | | |
| YMR103C | | |
| YMR105C | PGM2 | PhosphoGlucoMutase |
| YMR114C | | |
| YMR116C | ASC1 | Absence of growth Suppressor of Cyp1 |
| YMR135C | GID8 | Glucose Induced Degradation deficient |
| YMR140W | SIP5 | Snf1 Interacting Protein |
| YMR164C | MSS11 | Multicopy Suppressor of STA genes |
| YMR169C | ALD3 | ALdehyde Dehydrogenase |
| YMR173W | DDR48 | DNA Damage Responsive |
| YMR173W-A | | |
| YMR180C | CTL1 | Capping enzyme mRNA Triphosphatase-Like |
| YMR195W | ICY1 | Interacting with the CYtoskeleton |
| YMR196W | | |
| YMR210W | | |
| YMR240C | CUS1 | Cold sensitive U2 snRNA Suppressor |
| YMR250W | GAD1 | GlutAmate Decarboxylase |
| YMR251W-A | HOR7 | HyperOsmolarity-Responsive |
| YMR252C | | |
| YMR253C | | |
| YMR261C | TPS3 | Trehalose-6-Phosphate Synthase |
| YMR262W | | |
| YMR263W | SAP30 | SIT4 protein phosphatase Associated Protein |
| YMR280C | CAT8 | CATabolite repression |
| YMR291W | TDA1 | Topoisomerase I Damage Affected |
| YMR292W | GOT1 | GOlgi Transport |
| YMR297W | PRC1 | PRoteinase C |
| YMR304C-A | | |
| YMR316W | DIA1 | Digs Into Agar |
| YMR320W | | |
| YNL007C | SIS1 | SIt4 Suppressor |
| YNL036W | NCE103 | NonClassical Export |
| YNL042W | BOP3 | Bypass Of Pam1 |
| YNL069C | RPL16B | Ribosomal Protein of the Large subunit |
| YNL077W | APJ1 | Anti-Prion DnaJ |
| YNL093W | YPT53 | Yeast Protein Two |
| YNL097C | PHO23 | PHOsphate metabolism |
| YNL098C | RAS2 | homologous to RAS proto-oncogene |
| YNL115C | | |
| YNL120C | | |
| YNL144C | | |
| YNL155W | | |
| YNL156C | NSG2 | |
| YNL159C | ASI2 | Amino acid Sensor-Independent |
| YNL160W | YGP1 | Yeast GlycoProtein |
| YNL173C | MDG1 | Multicopy suppressor of Defective G-protein |
| YNL180C | RHO5 | Ras HOmolog |

| | | |
|---|---|---|
| YNL192W | CHS1 | CHitin Synthase |
| YNL194C | | |
| YNL195C | | |
| YNL200C | | |
| YNL251C | NRD1 | Nuclear pre-mRNA Down-regulation |
| YNL259C | ATX1 | AnTioXidant |
| YNL266W | | |
| YNL274C | GOR1 | GlyOxylate Reductase |
| YNL279W | PRM1 | Pheromone-Regulated Membrane protein |
| YNL305C | BXI1 | BaX Inhibitor |
| YNR001C | CIT1 | CITrate synthase |
| YNR002C | ATO2 | Ammonia (Ammonium) Transport Outward |
| YNR006W | VPS27 | Vacuolar Protein Sorting |
| YNR007C | ATG3 | AuTophaGy related |
| YNR014W | | |
| YNR034W | SOL1 | Suppressor Of Los1-1 |
| YNR049C | MSO1 | Multicopy suppressor of Sec One |
| YNR060W | FRE4 | Ferric REductase |
| YOL014W | | |
| YOL016C | CMK2 | CalModulin dependent protein Kinase |
| YOL024W | | |
| YOL032W | OPI10 | OverProducer of Inositol |
| YOL047C | | |
| YOL048C | RRT8 | Regulator of rDNA Transcription |
| YOL052C-A | DDR2 | DNA Damage Responsive |
| YOL083W | ATG34 | AuTophaGy related |
| YOL084W | PHM7 | PHosphate Metabolism |
| YOL089C | HAL9 | HALotolerance |
| YOL096C | COQ3 | COenzyme Q |
| YOL100W | PKH2 | Pkb-activating Kinase Homolog |
| YOL123W | HRP1 | Heterogenous nuclear RibonucleoProtein |
| YOL133W | HRT1 | High level expression Reduces Ty3 transposition |
| YOL150C | | |
| YOL151W | GRE2 | Genes de Respuesta a Estres (stress responsive genes) |
| YOL153C | | |
| YOL154W | ZPS1 | Zinc- and pH-regulated Surface protein |
| YOL162W | | |
| YOR003W | YSP3 | Yeast Subtilisin-like Protease III |
| YOR018W | ROD1 | Resistance to O-Dinitrobenzene |
| YOR019W | | |
| YOR023C | AHC1 | Ada Histone acetyltransferase complex Component |
| YOR026W | BUB3 | Budding Uninhibited by Benzimidazole |
| YOR027W | STI1 | STress Inducible |
| YOR028C | CIN5 | Chromosome INstability |
| YOR036W | PEP12 | carboxyPEPtidase Y-deficient |
| YOR041C | | |
| YOR049C | RSB1 | Resistance to Sphingoid long-chain Base |
| YOR052C | | |
| YOR054C | VHS3 | Viable in a Hal3 Sit4 background |
| YOR062C | | |
| YOR065W | CYT1 | |
| YOR068C | VAM10 | VAcuolar Morphogenesis |
| YOR089C | VPS21 | Vacuolar Protein Sorting |

| | | |
|---|---|---|
| YOR092W | ECM3 | ExtraCellular Mutant |
| YOR099W | KTR1 | Kre Two Related |
| YOR133W | EFT1 | Elongation Factor Two |
| YOR134W | BAG7 | |
| YOR137C | SIA1 | |
| YOR139C | | |
| YOR148C | SPP2 | Suppressor of PrP |
| YOR152C | | |
| YOR161C | PNS1 | pH Nine Sensitive |
| YOR173W | DCS2 | DeCapping Scavenger |
| YOR178C | GAC1 | Glycogen ACcumulation |
| YOR186W | | |
| YOR215C | AIM41 | Altered Inheritance of Mitochondria |
| YOR219C | STE13 | STErile |
| YOR220W | RCN2 | Regulator of CalciNeurin |
| YOR223W | | |
| YOR245C | DGA1 | DiacylGlycerol Acyltransferase |
| YOR267C | HRK1 | Hygromycin Resistance Kinase |
| YOR273C | TPO4 | Transporter of POlyamines |
| YOR289W | | |
| YOR317W | FAA1 | Fatty Acid Activation |
| YOR345C | | |
| YOR348C | PUT4 | Proline UTilization |
| YOR349W | CIN1 | Chromosome INstability |
| YOR374W | ALD4 | ALdehyde Dehydrogenase |
| YOR385W | | |
| YPL003W | ULA1 | Ubiquitin-Like protein Activation |
| YPL004C | LSP1 | Long chain bases Stimulate Phosphorylation |
| YPL014W | | |
| YPL017C | IRC15 | Increased Recombination Centers |
| YPL024W | RMI1 | RecQ Mediated genome Instability |
| YPL047W | SGF11 | SaGa associated Factor 11kDa |
| YPL054W | LEE1 | |
| YPL057C | SUR1 | SUppressor of Rvs161 and rvs167 mutations |
| YPL061W | ALD6 | ALdehyde Dehydrogenase |
| YPL062W | | |
| YPL064C | CWC27 | Complexed With Cef1p |
| YPL100W | ATG21 | AuTophaGy related |
| YPL123C | RNY1 | RiboNuclease from Yeast |
| YPL131W | RPL5 | Ribosomal Protein of the Large subunit |
| YPL154C | PEP4 | carboxyPEPtidase Y-deficient |
| YPL156C | PRM4 | Pheromone-Regulated Membrane protein |
| YPL159C | PET20 | PETite colonies |
| YPL186C | UIP4 | Ulp1 Interacting Protein |
| YPL201C | YIG1 | Yeast protein Interacting with Glycerol 3-phosphatase |
| YPL203W | TPK2 | Takashi's Protein Kinase |
| YPL219W | PCL8 | Pho85 CycLin |
| YPL221W | FLC1 | FLavin Carrier |
| YPL222W | FMP40 | Found in Mitochondrial Proteome |
| YPL223C | GRE1 | Genes de Respuesta a Estres (spanish for stress responsive genes) |
| YPL229W | | |
| YPL230W | USV1 | Up in StarVation |
| YPL238C | | |

| | | |
|---|---|---|
| YPL240C | HSP82 | Heat Shock Protein |
| YPL247C | | |
| YPL258C | THI21 | THIamine metabolism |
| YPL260W | | |
| YPL272C | | |
| YPL280W | HSP32 | Heat-Shock Protein |
| YPR005C | HAL1 | HALotolerance |
| YPR012W | | |
| YPR026W | ATH1 | Acid TreHalase |
| YPR030W | CSR2 | Chs5 Spa2 Rescue |
| YPR053C | | |
| YPR061C | JID1 | DnaJ protein Involved in ER-associated Degradation |
| YPR065W | ROX1 | Regulation by OXygen |
| YPR080W | TEF1 | Translation Elongation Factor |
| YPR127W | | |
| YPR149W | NCE102 | NonClassical Export |
| YPR154W | PIN3 | Psi+ INducibility |
| YPR155C | NCA2 | Nuclear Control of ATPase |
| YPR158W | CUR1 | Curing of [URe3] |
| YPR160W | GPH1 | Glycogen PHosphorylase |
| YPR184W | GDB1 | |
| YPR199C | ARR1 | ARsenicals Resistance |
| YER039C-A | | |
| YDR034W-B | | |
| YOR298C-A | MBF1 | Multiprotein Bridging Factor |
| YBR085C-A | | |
| YER053C-A | | |
| YNR034W-A | | |
| YDL085C-A | | |
